# Supplementary material for: ‘Maze’ not pathway: focus group exploration of patients’ and public experiences of the UK NHS elective total joint arthroplasty pathway
Source: BMJ Open. 2023 Aug 9;13(8):e066762. doi: 10.1136/bmjopen-2022-066762 (PMC10414077; doi:10.1136/bmjopen-2022-066762)
Supplement: Supplementary data [file bmjopen-2022-066762supp001.pdf]

SUPPLEMENTAL MATERIAL

Online Supplementary File 1: SUMMARY TOPIC GUIDE 1 – GROUPS 1 & 2 (PATIENTS)

SECTION 1: EXPLORING PRACTICE: BEFORE, DURING AND AFTER SURGERY

Looking at this picture of the patient journey, we want to find out what was important to you at different stages:

1. **Before surgery** (orange box):

   At the point at which you were deciding whether to have a hip / knee replacement operation, what was important for you to know about?

2. **In hospital** (pink box):

   Based on your experience of having planned joint surgery in the NHS:

3. **After surgery** (green box):

   Once at home and following surgery, did you need to contact the NHS hospital where you were treated for any reason?

SECTION 2: EXPLORING GIRFT PROGRAMME PRIORITIES

4. In the presentation, we talked about how the GIRFT programme is trying to help hospitals across England provide similar 'results' for all patients who undergo planned orthopaedic surgery (e.g. to improve their life and reduce pain).

5. Are there any GIRFT programme priorities that were covered in the presentation today that we have not discussed and are also important to you?

6. Is there anything else important to you about joint surgery in the NHS, different from the GIRFT programme priorities and that we haven't talked about today?

SECTION 3: CLOSING COMMENTS

7. Finally, if you could give some feedback to the people leading this programme – what might you say?

Online Supplementary File 2: SUMMARY TOPIC GUIDE 2 – GROUP 3 (PUBLIC INDIVIDUALS)

SECTION 1: EXPLORING PRACTICE: BEFORE, DURING AND AFTER SURGERY

Looking at this picture of the patient journey, we want to find out what is important to you at different stages:

1. **Before surgery** (orange box):

   Scenario: Your GP has recommended that you see an orthopaedic surgeon, as you might need joint replacement surgery (e.g. a hip or knee replacement)

2. **After surgery** (green box):

   Scenario: Imagine you are at home after your hip/ knee replacement operation, and you become concerned about your recovery:

SECTION 2: EXPLORING GIRFT PROGRAMME PRIORITIES

3. In the presentation, we talked about how the GIRFT programme is trying to help hospitals across England provide similar 'results' for all patients who undergo planned orthopaedic surgery (e.g. to improve their life and reduce pain).

4. Are there any GIRFT programme priorities that were covered in the presentation today that we have not discussed and are also important to you?

5. Is there anything else important to you about joint surgery in the NHS, different from the GIRFT programme priorities and that we haven't talked about today?

SECTION 3: CLOSING COMMENTS

6. Finally, if you could give some feedback to the people leading this programme – what might you say?

Jasim S, *et al. BMJ Open* 2023; 13:e066762. doi: 10.1136/bmjopen-2022-066762

Online Supplementary File 3: DETAILED STATEMENT FOR REFLEXIVITY

Reflexivity relates to acknowledgment of sensitivity to the ways in which the researchers and the research process may have shaped the data collected, including the role of prior assumptions and experience.[1]

Wider context of the research

Each focus group began with a short presentation explaining the context of the wider 'Getting It Right First Time' (GIRFT) [2] programme in elective orthopaedic surgery in England, and the programme priorities, to ensure all participants had a minimum level of knowledge. During analysis and interpretation, this pre-information was considered, and the possibility of how this knowledge might have shaped what was said.

Professional background of the research team

Members of the research team involved in fieldwork considered the ways in which their interactions with participants might be influenced by their own professional background, experiences, and prior assumptions. Co-facilitators (SJ and JL) were both academic research fellows from non-clinical backgrounds, the patient advisor (RM) who also co-facilitated focus groups had a non-clinical background but had previous experience of non-orthopaedic surgery. The observers (FA and HB) were both senior academic researchers who had clinical backgrounds. During our analysis and interpretation stages we considered whether knowing about our professional backgrounds could have impacted on participants' willingness to openly talk and share their experiences.

Potential for harm or distress

Members of the research team involved in fieldwork were sensitive to the possibility that focusing on the research topic (i.e. recalling previous surgical / in-hospital experiences) could potentially provoke anxiety in the focus group participants concerning the disclosure of adverse events. At the start of each focus group, co-facilitators (RM, SJ, JL) ensured that focus group participants could step out at any time or reveal or disclose as much of their previous experiences and perspectives as they felt comfortable with. At the end of each focus group, the research team stayed behind in the room to ensure that participants had the opportunity to seek support if they were feeling distressed by their participation. No focus group participants expressed such concerns or appeared to be distressed or uneasy.

Collaboration in knowledge production

Collaborative research is valued for its ability to bring together multiple researchers with distinctive and specialist perspectives to tackle large or complex research problems. There was a strong commitment within the research team from the outset to work collaboratively in the collection,

Jasim S, *et al. BMJ Open* 2023; 13:e066762. doi: 10.1136/bmjopen-2022-066762

analysis, interpretation and reporting of the qualitative data, though individual involvement with the various stages of the research process varied as necessary. Team members closely involved in fieldwork (SJ, JL, RM, FA, HB) met frequently on separate occasions to discuss the progress of fieldwork and reflect on data collection. Meetings intensified during the initial stages of data collection and early analysis, when the topic guides and focus group materials were being produced and piloted, and themes and codes were beginning to be identified. At these crucial stages, input was sought from a senior member of the research team with extensive experience of qualitative research (NJF). In the final stages of analysis, input was sought from another senior member of the research team to sense check the coherence, truthfulness, and accuracy of the findings (RR). This resulted in an analytic strategy that was informed by insights from team members with a broad understanding of the research field and methodological issues, and those with field-based contextual and experiential understanding.

Jasim S, *et al. BMJ Open* 2023; 13:e066762. doi: 10.1136/bmjopen-2022-066762

REFERENCE LIST

1. Jootun D, McGhee G, Marland G R. Reflexivity: promoting rigour in qualitative research. *Nurs stand.* 2009;23(23): 42-47.
2. Briggs T. Getting it Right First Time: improving the quality of orthopaedic care within the National Health Service in England, 2012. London, United Kingdom: British Orthopaedic Association.
