# Supplementary material for: ‘Maze’ not pathway: focus group exploration of patients’ and public experiences of the UK NHS elective total joint arthroplasty pathway
Source: BMJ Open. 2023 Aug 9;13(8):e066762. doi: 10.1136/bmjopen-2022-066762 (PMC10414077; doi:10.1136/bmjopen-2022-066762)
Supplement: Author's manuscript [file bmjopen-2022-066762.draft_revisions.pdf]

BMJ Open is committed to open peer review. As part of this commitment we make the peer review history of every article we publish publicly available.

When an article is published we post the peer reviewers' comments and the authors' responses online. We also post the versions of the paper that were used during peer review. These are the versions that the peer review comments apply to.

The versions of the paper that follow are the versions that were submitted during the peer review process. They are not the versions of record or the final published versions. They should not be cited or distributed as the published version of this manuscript.

BMJ Open is an open access journal and the full, final, typeset and author-corrected version of record of the manuscript is available on our site with no access controls, subscription charges or pay-per-view fees (http://bmjopen.bmj.com).

If you have any questions on BMJ Open's open peer review process please email info.bmjopen@bmj.com

BMJ Open

‘Maze’ not pathway: patients’ and public experiences of the UK NHS elective total joint arthroplasty pathway.

Journal:	*BMJ Open*
Manuscript ID	bmjopen-2022-066762
Article Type:	Original research
Date Submitted by the Author:	16-Sep-2022
Complete List of Authors:	Jasim, Sarah; The London School of Economics and Political Science, Care Policy and Evaluation Centre<br>Aspinal, Fiona; University College London, Department of Applied Health Research<br>Mehta, Raj; University College London, Department of Applied Health Research<br>Ledger, Jean; University College London<br>Raine, Rosalind; University College London, Institute of Epidemiology & Health<br>Fulop, Naomi; University College London, Applied Health Research<br>Barratt, Helen; UCL, Department of Applied Health Research
Keywords:	Orthopaedic & trauma surgery < SURGERY, QUALITATIVE RESEARCH, Hip < ORTHOPAEDIC & TRAUMA SURGERY, Knee < ORTHOPAEDIC & TRAUMA SURGERY, Quality in health care < HEALTH SERVICES ADMINISTRATION & MANAGEMENT

SCHOLARONE™
Manuscripts

I, the Submitting Author has the right to grant and does grant on behalf of all authors of the Work (as defined in the below author licence), an exclusive licence and/or a non-exclusive licence for contributions from authors who are: i) UK Crown employees; ii) where BMJ has agreed a CC-BY licence shall apply, and/or iii) in accordance with the terms applicable for US Federal Government officers or employees acting as part of their official duties; on a worldwide, perpetual, irrevocable, royalty-free basis to BMJ Publishing Group Ltd ("BMJ") its licensees and where the relevant Journal is co-owned by BMJ to the co-owners of the Journal, to publish the Work in this journal and any other BMJ products and to exploit all rights, as set out in our licence.

The Submitting Author accepts and understands that any supply made under these terms is made by BMJ to the Submitting Author unless you are acting as an employee on behalf of your employer or a postgraduate student of an affiliated institution which is paying any applicable article publishing charge ("APC") for Open Access articles. Where the Submitting Author wishes to make the Work available on an Open Access basis (and intends to pay the relevant APC), the terms of reuse of such Open Access shall be governed by a Creative Commons licence – details of these licences and which Creative Commons licence will apply to this Work are set out in our licence referred to above.

Other than as permitted in any relevant BMJ Author's Self Archiving Policies, I confirm this Work has not been accepted for publication elsewhere, is not being considered for publication elsewhere and does not duplicate material already published. I confirm all authors consent to publication of this Work and authorise the granting of this licence.

For peer review only

‘Maze’ not pathway: patients’ and public experiences of the UK NHS elective total joint arthroplasty pathway.

*Sarah Jasim, Care Policy and Evaluation Centre, London School of Economics and Political Science, Houghton Street, Holborn, London, WC2A 2AE, UK s.jasim@lse.ac.uk

Sarah Jasim, Care Policy and Evaluation Centre, London School of Economics and Political Science, UK

Fiona Aspinal, Department of Applied Health Research, University College London, UK

Raj Mehta, Patient Advisor, NIHR ARC North Thames Research Advisory Panel, Department of Applied Health Research, University College London, UK

Jean Ledger, Department of Applied Health Research, University College London, UK

Rosalind Raine, Department of Applied Health Research, University College London, UK

Naomi J. Fulop, Department of Applied Health Research, University College London, UK

Helen Barratt, Department of Applied Health Research, University College London, UK

*corresponding author

Word count: 2934 words

For peer review only

ABSTRACT

Objective: To explore patient and public perceptions of planned improvements to the NHS TJA pathway.

Design: Three qualitative focus groups undertaken March – May 2018, as part of a mixed-methods evaluation of GIRFT. Transcripts were subject to framework analysis to identify thematic content between October 2018 – October 2021.

Setting: Elective TJA surgery in the English NHS.

Participants: Two focus groups including patients who had undergone TJA in the previous two years (Group 1: n=5; Group 2: n=4) and the other individuals who had not but were aged 60+ (Group 3: n= 5). Participants were recruited via community groups and patient panels.

Results: Participants' perspectives were categorised into themes and mapped on to stages of the TJA pathway. 14 individuals took part in the focus groups; all were aged over 60; 50% were female; and 9 (64%) had undergone TJA surgery. Although perioperative care is often the focus of improvement efforts, the patient journey begins when individuals present to primary care with joint pain. Consequently participants had concerns about other aspects of the pathway, such as obtaining a surgical referral. Pre-referral interventions aimed at potentially avoiding the need for surgery (i.e. physiotherapy) were perceived as restricting access to secondary care. Patient experience was also conceptualised as a 'maze', rather than the logical, sequential process set out in clinical guidelines; exacerbated by a lack of information, knowledge, and power imbalances.

Conclusion: The linear conceptualisation of the TJA pathway is at odds with patient experience. Improvement programmes focussed on perioperative care fail to consider their concerns and priorities. Patients should be directly involved in improvement programmes, to ensure that patient experience is optimised, as well as processes and outcomes of care.

For peer review only - http://bmjopen.bmj.com/site/about/guidelines.xhtml

STRENGTHS AND LIMITATIONS OF THIS STUDY

- This is the first study to look at the entire TJA pathway from the perspective of patients and the public and explore how their priorities differ from those of clinicians and managers seeking to improve care.
- This work is part of the first independent evaluation of the high profile, national improvement programme, Getting it Right First Time (GIRFT).
- Co-producing research with a patient advisor ensured the patient perspective was central to our work from planning through to analysis and dissemination.
- We sought to recruit via a wide range of different channels, including professional societies, patient groups and local community groups (including multi-faith organisations).
- Nevertheless we were not able to recruit a diverse sample with respect to ethnicity, and one participant withdrew because of fears of cultural stigma.

For peer review only

For peer review only - http://bmjopen.bmj.com/site/about/guidelines.xhtml

BACKGROUND

Elective hip and knee replacement are the two most common total joint arthroplasty (TJA) procedures, and two of the most frequently performed and effective surgical procedures in the UK [1,2] and globally.[3] With an increasingly ageing population, and trend for TJA earlier in life,[4] the predicted growth in global demand is likely to have a significant impact on health systems,[5] such as the National Health Service (NHS) in England, which already faces a large backlog of care following the COVID-19 pandemic.[6]

A care pathway [7] is a multidisciplinary tool for planning the care of specific, well-defined groups of patients who have a predictable clinical course, such as TJA. Drawing on research evidence and best practice, it sets out how different components of care should be optimised and sequenced, to enhance quality [8] and improve patient safety. This approach has been shown to reduce unnecessary variation [7,9-12] and may improve patient outcomes and optimise resource use.[7,13-15] Standardisation of care is important for national improvement programmes such as the English 'Getting It Right First Time' programme (GIRFT), which aims to tackle unwarranted variation in orthopaedic surgery across the NHS.[16] GIRFT sought to develop a 'best of the best' clinical pathway [17] to improve patient experience and expedite recovery following TJA. Although clinical perspectives are central to pathway development,[9] the views of current and future patients who must navigate them, are equally important to optimise experience, outcomes, and efficiency.[18]

In the UK, general practitioners (GPs) act as gatekeepers to secondary care, including the TJA pathway. Patients experiencing hip or knee pain typically first present to GP. In some English regions, local commissioners (leaders responsible for purchasing health care on behalf of local communities), are increasingly setting criteria that patients must meet before they can be referred to an orthopaedic surgeon. Current guidance recommends conservative management initially, for at least three months, with the aim of potentially avoiding the need for the patient to undergo surgery and all that that entails. Measures include medication, physiotherapy, and / or support with lifestyle and weight loss. If this is not successful, the GP should refer the patient to an orthopaedic surgeon to discuss potential surgery.

Existing research about patient perspectives of the TJA pathway has tended to focus on patient satisfaction and experience [19] at specific stages of the pathway, such as deciding to undergo surgery,[20] pre-operative education,[21] waiting for surgery,[22-23] and management of post-operative pain.[13] Given shared decision-making is considered integral to care pathways as well as wider efforts to improve care, there is currently no evidence on how patients and public view planned improvements to the TJA pathway, and whether their priorities align with those of clinicians and managers.[25-26] This study sought to address that gap by exploring patient and public perceptions of planned improvements to the TJA pathway, including the factors that influenced this. The research was part of a wider mixed methods evaluation of the GIRFT programme.[16, 27]

For peer review only

METHODS

Study Design and Recruitment

Focus groups enabled us to collect views from multiple participants simultaneously and explore meanings behind those views, which were generated, discussed, and refined through group interactions in a way that could not happen with other methods.[28]

We sought to recruit between four to eight participants to each of three focus groups, based on evidence regarding the optimal number of groups and participants for focus group research.[29] Groups 1 and 2 included patients who had undergone an elective hip or knee replacement in the previous two years (March 2016–March 2018). Group 3 included individuals who had not undergone TJA but were in the age bracket most likely to receive this type of intervention (age 60+) (Figure 1).[24] This enabled us to compare experiences of those who had undergone TJA with the expectations of those who had not, and how this influenced participants' views. In this paper we report the findings from all three groups together, referring to individuals as participants, except where there were clear differences between those who had undergone surgery, and those who had not. We recruited participants via community organisations across London (UK) whose membership was likely to be aged 60 and over (e.g. third sector organisations, day centres and activity clubs). We also invited participation from eligible patient advisory group members from relevant organisations (e.g. the British Orthopaedic Association). Researchers (SJ, JL) conducted telephone and e-mail screening and collected preliminary demographic information to maximise sample diversity (Table 1).

Table 1: Participant characteristics

	**Group 1**<br>(Patients – n=5)	**Group 2**<br>(Patients – n=4)	**Group 3**<br>(Public – n=5)
**Sex**	Male (n=2), Female (n=3)	Male (n=2), Female (n=2)	Male (n=3), Female (n=2)
**Age range**	60-69 (n=2)<br>70-79 (n=3)	60-69 (n=1)<br>70-79 (n=1)<br>80-89 (n=2)	60-69 (n=3)<br>70-79 (n=2)
**Ethnicity**	White British (n=4)<br>White European (n=1)	White British (n=3)<br>White South African (n=1)	White British (n=4)<br>White American (n=1)

For peer review only

Data Collection

We conducted three focus groups between March to May 2018 in an academic (non-healthcare) setting. Observation data were collected by senior researchers (FA or HB) to capture any contextual factors that might have impacted data quality, such as the topics being discussed or depth of discussion. All participants gave informed consent. Each focus group began with a brief presentation by the research team, providing a high-level overview of efforts to improve the TJA pathway for patients, including GIRFT.[16]

Focus group discussions were informed by semi-structured topic guides (online supplementary file 1-2), which included participants' priorities before, during and after surgery, as well as their views of the planned improvements to the TJA pathway. Each group lasted approximately two hours and was audio-recorded using an encrypted device for professional transcription in full.

Patient and Public Involvement

Three focus groups were co-facilitated by a researcher (SJ or JL) and the patient advisor (RM).[30] Following advice from our patient advisor (RM),[30] we (SJ) also provided participants with a simplified illustration of the UK TJA care pathway (Figure 2), based on clinical guidance at the time.[31] The pathway diagram and topic guides were piloted with a research advisory panel of patient advisers and refined initially, and then iteratively as the research progressed (i.e., diagram title and emphasis of questions), to take account of emerging findings, in line with accepted qualitative methods.[32] Preliminary results of the study were verbally presented to participating patient advisors.

Data Analysis

The core research team (HB, FA, RM, SJ, JL) held de-brief meetings after each focus group to ensure triangulation between focus group findings and researcher observations. Observation notes and field notes were not subject to formal analysis but were used in debrief and analysis meetings to ensure transcript data were contextualised. Transcript data were analysed using a Framework approach.[33] We used the care pathway diagram (Figure 2) and structure of the topic guides (online supplementary file 1-2) to develop the initial coding framework, and incorporated concepts and meanings identified in the data.[14]

The first stage of analysis involved familiarisation, where researchers (SJ, JL, FA, HB) read and collaboratively reviewed transcripts to understand meanings, concepts, and ideas, in a reflexive manner (see online supplementary file 3). The wider research team (SJ, JL, RM, FA, HB, NJF) then identified and confirmed parameters of the main themes to form the basis of a thematic framework in a series of separate meetings. Data were indexed based on the thematic framework using MAXQDA software (SJ). Indexed data were organised into thematic charts for each category, in Microsoft Excel, using direct quotations where possible. These were reviewed and modified when necessary to clarify meaning (FA). Themes were further mapped and interpreted to understand areas of significance to patients and the public (SJ, FA, HB, JL, RM, NJF, RR).

Ethics

Ethical approval was granted by NHS North West - Liverpool East Research Ethics Committee (16/NW/0654).[16]

For peer review only - http://bmjopen.bmj.com/site/about/guidelines.xhtml

RESULTS

Participants

Seventeen individuals agreed to participate, although three withdrew on the day. Two because of heavy snowfall (Group 2 and Group 3) and one due to fears of cultural stigma from openly discussing experiences (Group 2). All 14 participants were aged over 60 and all self-identified as White British or White Other. 50% were female, and 9 participants had undergone TJA surgery in the previous two years. Of the 5 who had not undergone surgery (i.e. Group 3), 3 had friends or family who had previously undergone TJA..

Our original goal was to explore participants' views about planned improvements to the TJA pathway (such as GIRFT),[2] which were focused particularly on the perioperative 'in-hospital' (Figure 2) phase of the care pathway. However, it quickly became apparent that other phases of the pathway were of equal, if not more, importance to participants. Participants understood the pathway diagram, but argued that whilst the streamlined, linear presentation may be how it appears to clinicians and managers, it did not reflect their experience. We therefore describe here participants' views about priorities for improvement; the realities of the TJA pathway; and barriers and facilitators they encountered whilst navigating the pathway (participant quotations are provided in Table 2).

For peer review only

Table 2: Focus group (FG) quotes

Ref.	FG No.	Gender	Quote
Quote1	1	Male	'…… that's [referring to the first part of the pathway diagram] probably where most problems, as far as I'm concerned, arose. In that trying to see a consultant was very difficult.'
Quote2	1	Male	'…I get a letter in the post saying, it's a confirmation of the date, so that would be followed by another letter that says they've just cancelled that appointment…With no explanation, it just says it's cancelled… "What happens, where are we now, or what happens now?"…they said, "Oh well, it's got to go in before the assessment committee"… Well, this is very, very … upsetting that… I'm in a lot of pain, I want to see a consultant, and there's all these obstacles in the way before you can even see the consultant.'
Quote3	1	Female	'I had months of physiotherapy right through till February the following year. It wasn't helping, I didn't think it was helping a lot... And then in the February I spoke to my physiotherapist, asked him… Am I going to go on and on like this? … So, I'm now thinking, "…they're just… shuffling me along," obviously … would I like to just go ahead with the op…'
Quote4	1	Male	'Yeah, I think it's a postcode lottery, isn't it, where some people live that there's a huge waiting list and I think the GPs…refer them to the physiotherapist first so… keeps them out of the way for a bit…'
Quote5	2	Male	'…the GP is the stopping point, and it causes a lot of the other things going up the line to be worse than they need to be because they resist. Obviously, they're the Clinical Commissioning Group that actually drives them on this and say, 'Don't refer, don't refer, don't refer, we'll even give you some extra bonus points if you don't refer.'
Quote6	1	Mixed	Male participant: 'But I paid for this, it wasn't NHS, I paid for the treatment. It was within six months of after the NHS, so the NHS treatment was still fresh in my mind, and so I was going through the private sector. And I was interested to compare it, and if you ask me which one, I preferred, it would be the NHS. I'm not impressed with private treatment at all.'<br><br>Female participant: 'Why did you decide to pay for it?'<br><br>Male participant: 'Because I didn't want to go through all the procedure we had before trying to get an appointment… And it developed again very quickly and, you know, I want to get on with life, I don't want to be hanging around waiting for operations, so …'
Quote7	2	Male	'…so it was down to you to look after your own, and when it all starts to go wrong, you've got to go to your GP and you've got to persuade him to refer you to a specialist…'
Quote8	1	Female	'…I felt I had to bully them in order to have the surgery…'
Quote9	1	Female	'This is elective, yeah, which means you're making the choice, so surely it should be an informed choice.'
Quote10	1	Female	'– I was just taken aback by how much I felt like a widget on a production line.'
Quote11	3	Female	'…if you like, you'd need a referral to Social Services. I think that should be part of the pre-operative planning rather than suddenly realising afterwards. What I was talking about was the OT assessment and the follow-up physiotherapy, I wasn't talking about Social Services, about social care support, which you may well need and that should have really been part of the planning beforehand. Because if you've got to wait 'til afterwards you'll be in hospital for a month before anything happens. I know because I've been a social worker so, you know, I know what it's like.'
Quote12	1	Female	'lack of communication between various people, the doctors, the nurses, so that I felt I was the person who knew more than anyone else about what was going on, but I knew least about what should be going on'
Quote13	3	Female	'I'd like to know about physiotherapy and after care and the system they use…So I'd want to know, if I was going to go in, whether I'd be not only would the surgery be good and efficient but there would be a very good programme coming out in after care and who'd be doing that with me.'
Quote14	3	Female	'I want to come back to the Social Services and what happens when you get out of hospital, especially if you live alone or... you know, how do you manage for that period of time after hospital, that's an important handover and that's not always handled really well.'

For peer review only - http://bmjopen.bmj.com/site/about/guidelines.xhtml

Quote15	1	Female	'I mean this is big problem, I think, that I mean everybody knows that it's a big problem with social care, but you know, I said to the nurse that was asking me about it, you know, "What do people do if they haven't got family to help?" and she sort of shook her head sadly.'
Quote16	1	Male	'..like a maze'
Quote17	1	Male	'all the time there's never any communication as to what's happening. So, you're left in the dark, and that itself is upsetting because, you know, you want to get it dealt with, you don't know at what stage it's going to happen, and you're just being pushed from pillar to post, and nobody will give you any information.'
Quote18	3	Female	'There's a big thing about sort of communication through the line so that people don't rely on you as the rather disorientated patient after an operation to actually be the person who's informing so that there are other people sharing information, but you've also got a voice, that your voice is going to be heard or that you've elected someone to speak for you and that person's voice is going to be heard as well.'
Quote19	3	Male	'…one of the dangers of the information revolution… a lot of information is totally unhelpful…'
Quote20	3	Male	'information tends to be... You're always given something when you go out - and this is all kinds of hospitalisation, not just surgery… but it's never quite enough… but it's generally not good…'
Quote21	1	Female	'because I can see from this chart [diagram] that I'm on a conveyor belt and that's the feeling that I had. And actually, I felt that I'd been in the dark from the start. And I felt that there is information there if I ask the right question, but I don't know what question to ask. And I have felt that I have to look it up a lot myself, I have to find out things myself'
Quote22	2	Female	"Well, personally I wasn't aware that there was even any choice."
Quote23	3	Female	'It is actually a plus point for hospitals to make their statistics available publicly in some way because people will search for excellence and people will avoid mediocrity, and I certainly don't depend on my GP for that...'
Quote24	1	Female	'So, that question of choice actually goes all the way through. Is it really choice or not and how much power do we really have? How much knowledge do we have?'
Quote25	1	Female	"I was so intimidated by him, and so overwhelmed by him, I thought, 'Right, okay, well, you know, I'm just going to … there's no point in my doing anything else here'."
Quote26	3	Female	'…care-givers are important too and I can't tell you how many times I was at a doctor and the doctor just ignored the questions that I had to ask because I was not the patient...'
Quote27	1	Female	'But it was about two weeks before my operation, so if you are anxious and you feel like you're in the dark, that's a long time to wait before you get those questions, a chance to answer those questions, and you find out new information. It was at the joint school that I found out that you don't have a general anaesthetic, that you have a spinal anaesthetic.'
Quote28	1	Female	'He didn't even say to me that there is a hip replacement school and that you will get to attend the school before the operation. He could have said that to me. And that, you know, sometime down the line, all your questions will be answered…'
Quote29	2	Male	'I did have the information session… but there was very little emphasis on pre-hab. I knew that pre-hab was a very critical issue in the recovery, and I did it, I went to a private physio, got some guidance, some practical guidance about exercises and whether I was doing them right. And I think this is something that the NHS does not give enough emphasis to.'
Quote30	1	Female	'But then physio told me that there was a system whereby you get a choice of hospitals for having your operation in. I didn't know that either. She said, 'Haven't you heard about this before?' And she gave me a list of six hospitals, the physio… she said, 'Look them up, go and read the CQC report on these hospitals and see which one you like, and if you prefer one of the other ones, come back and let me know'. I'm sorry, having come through all this and been so in the dark I'm now being told to decide on what hospital.'
Quote31	1	Female	'I knew the system more, I was much more able to work my way through it, to talk to the right people, to make the decision'
Quote32	3	Female	'…I'd want the occupational therapist to assess me, assess my home, work out what the … support I'm going to get at home to actually help me achieve mobility and how accessible they are…'

For peer review only

For peer review only - http://bmjopen.bmj.com/site/about/guidelines.xhtml

Patient priorities: obtaining a referral to see an orthopaedic surgeon

Although the peri-operative phase of the pathway is often the focus of improvement programmes, such as GIRFT, individuals who had undergone TJA argued that the challenge of obtaining a referral to an orthopaedic surgeon is in fact a greater concern (see Table 2, Quote1). This was 'upsetting' given their level of pain or disability (Quote2). Some participants found that GP-recommended non-surgical interventions resulted in little or no improvement, whilst others had been in too much pain to engage with physiotherapy (Quote3). Consequently, they viewed this approach simply as a mechanism for managing demand (Quote4). Some participants believed that GPs had pressure placed on them to not refer patients to secondary care (Quote5, Quote6). Others felt that external pressures to ration NHS services impacted their access, such as referrals being reviewed by an 'assessment committee' (Quote2) or appointments being delayed by repeated, unexplained cancellations.

Because of the obstacles they had faced, participants spoke about the difficulties encountered to get onto the pathway, and secure an orthopaedic referral, and in some cases, 'persuade' (Quote7) or even 'bully' (Quote8) healthcare professionals, along the way whilst on this 'conveyor belt' (Quote9) or 'production line' (Quote10).

The realities of the elective TJA pathway

Participants, especially those who had not undergone surgery (Group 3), emphasised the importance of joined-up care planning throughout the pathway, including clinical teams planning surgery dates to coincide with available post-operative support (Quote11). Disconnected communication and ways of working between health providers at different stages of the pathway, left patients (Groups 1 and 2) having to assume responsibility for their own care, despite feeling powerless with insufficient information to make such informed decisions (Quote12). The lack of joined-up care was important to all groups, further worsened by poor communication. For example, they described poor discharge procedures where communication with wider services was lacking, such as pharmacy, community health services and social care (Quote14, Quote15). Care pathways did not end at discharge from secondary care but continued into the rehabilitation period, which was also important to all participants (Quote13).

Navigating the 'maze'

Without joined-up, forward-planning, patients had to manage and navigate a range of complex local health systems alone. They were left feeling adrift from the system, and many participants felt they were ill-equipped to tackle the obstacles they encountered because of asymmetries of both information and power. As one neatly summarised, the pathway is 'like a maze' (Quote16).

Lack of information and knowledge

Participants reported that from the point of referral from primary care, they were 'left in the dark' (Quote17, Quote9). A lack of communication (Quote18) or appropriate, timely information from their healthcare provider hindered their understanding of the process (Quote17, Quote19, Quote20) making it difficult for them to make 'informed choices' (Quote9). This was compounded for some by a lack of knowledge, meaning that they felt they did not know what questions they should be asking. This was particularly a concern in the stages prior to surgery but differed according to participants' direct or indirect experiences; location of care; and pre-existing knowledge or professional background (Quote21).

For peer review only - http://bmjopen.bmj.com/site/about/guidelines.xhtml

Although clinical guidance[17] implies shared decision-making should be built into the pathway, participants' knowledge about available choices varied. For example, some participants in all groups had not known that they could choose the hospital they went to, or their surgeon, whilst others had sought out information to inform their choice (Quote22, Quote23). Nevertheless, even those who were aware that they could exercise choice, felt that they lacked sufficient knowledge to do so (Quote24).

Power imbalance

Alongside a lack of information and communication, many participants described how they had to disrupt existing power dynamics to navigate the TJA pathway. Asymmetries of power in relationships with key health professionals could significantly impact their experience of care (Quote10) and their progress along the pathway. For example, negative interactions with a surgeon had halted one participant's journey during their first appointment with a surgeon and hindered their access to pathway at the very start (Quote25). Another participant, who had cared for a TJA patient, described how her attempts to advocate for them were ignored (Quote26).

Overcoming obstacles

Participants described three distinct sources of knowledge which they drew on to differing degrees to overcome the obstacles they faced: formal learning; informal learning; and lived experience.

Formal learning

Formal learning opportunities offered to patients prior to surgery had helped some. For example, pre-operative education groups (also known as 'Joint Schools') run by hospital staff for patients listed for surgery, had alleviated fears, increased knowledge, and provided an opportunity to ask questions. Nevertheless, for some, the programme was offered very close to the date of the procedure, meaning that they had a long wait to raise their concerns. Consequently some would have liked this to have been offered much earlier in the pathway (Quote28).

Informal learning

Some participants also sought out or expected to seek out information for themselves that they thought they needed to navigate the process. This included details about medication (carer, Group 3); chasing appointments (female patient, Group 1); and undertaking their own research to inform choices (all groups) and guide decision-making (female patient, Group 1). Others had also sought information from health professionals such as physiotherapists who were familiar with the TJA pathway, which helped them feel 'less in the dark' (Quote30).

Lived experience

Participants who were able to draw on a previous experience of undergoing surgery, used this to help inform choices and decision-making. Consequently they felt better able to navigate the pathway on subsequent occasions. Those who had previously undergone a TJA knew to request additional information about regaining function, resuming activities, and exercising, not only to improve outcomes but also to assist navigating the crucial post-operative stage of the pathway (Quote32). Some also believed that this knowledge had enabled them to expedite the process on subsequent occasions (Quote6), including identifying potential short cuts (Quote31, Quote7).

For peer review only - http://bmjopen.bmj.com/site/about/guidelines.xhtml

DISCUSSION

Despite ongoing efforts to streamline and standardise care, patient experience differs significantly from the seemingly logical, sequential process set out in clinical guidelines. Importantly for patients, the pathway does not begin and end at the doors of the hospital. For example, it starts at first presentation to primary care with joint pain. A lack of timely information and communication also leaves patients feeling that they have been 'left in the dark' to navigate a 'maze'. This sense is exacerbated by power asymmetries between patients and clinicians. Whilst formal education programmes such as 'Joint School' can be helpful, many patients have to seek out additional information for themselves to answer their queries; understand the procedure and navigate the TJA pathway.

Previous research has largely focused on specific phases of the care pathway. This is the first study to look at the entire TJA pathway from the perspective of patients and the public and explore how their priorities differ from those of clinicians and managers seeking to improve care. Co-producing research with a patient advisor ensured the patient perspective was central to our work from planning through to analysis and dissemination. As socio-economic circumstances may impact a patient's decision to undergo TJA,[34] we sought to recruit via a wide range of different channels, including professional societies, patient groups and local community groups (including multi-faith organisations). Nevertheless we were not able to recruit a diverse sample with respect to ethnicity. Indeed, one participant withdrew because of fears of cultural stigma.

Our findings have several implications for clinicians and policymakers. Although perioperative care is often the focus of clinically-led efforts (e.g. GIRFT) [2] to improve the TJA pathway, participants also emphasised that obtaining a referral to a surgeon is often the greatest challenge, and the post-operative period following surgery is a priority. We have highlighted that non-surgical interventions (e.g. physiotherapy) prior to surgical referral are perceived as a means of managing demand. While this remains the case, as well as potentially damaging patient trust, it is likely to impact patient engagement with evidence-based care. Efforts to provide patients with information about their procedure and likely recovery (e.g. via 'Joint Schools') are welcome, but many want information about the pathway earlier than this, so that they are not left feeling ill-equipped to navigate the 'maze' of care themselves, especially if they have not previously undergone surgery. At a policy-level, those working to standardise services using care pathways should also be aware that patient experience is seldom analogous to their linear, stepwise conceptualisations. In line with the existing literature, our findings suggest that experience-based co-design should be a core part of pathway re-design.[35]

CONCLUSION

In conclusion, we have highlighted the disparity between the priorities of improvement programmes such as GIRFT and those of patients and the public. Whilst improvement programmes typically seek to improve peri-operative care, patient concerns lie elsewhere, for example obtaining a referral to an orthopaedic surgeon. The typically linear conceptualisation of TJA pathways in policy documents is also at odds with how patients experience the process. With rates of TJA set to increase globally, patients and public must be directly involved in improvement programmes, to optimise not only processes and outcomes of care, but also patient experience.

For peer review only - http://bmjopen.bmj.com/site/about/guidelines.xhtml

DECLARATIONS

Acknowledgements: The authors would like to acknowledge and thank the focus group participants, and especially Steven Towndrow, Wendy Chandler, Stephanie Hume, Patricia Hallam and the NIHR ARC (formerly CLAHRC) North Thames Research Advisory Panel patient advisers for their efforts in preparation and organisation of the focus groups.

Author Contributions: HB was principal investigator. HB and RR initiated the research. HB, NJF and RR designed the evaluation. SJ, RM and JL were responsible for qualitative data collection; SJ, FA,RM, JL, NJF and HB analysed the qualitative data. SJ, FA, RM, JL, RR, NJF and HB drafted the manuscript and all authors contributed to reviewing and substantive revision. All authors approved the final version. All authors had full access to all the data in the study and accept responsibility to submit for publication. HB will act as guarantor.

Funding: This reports independent research funded by the National Institute for Health Research (NIHR) Applied Research Collaboration (ARC) North Thames (award/grant number is not applicable).

Disclaimer: This report is independent research funded by the National Institute for Health Research ARC North Thames. The views expressed in this publication are those of the authors and not necessarily those of the National Institute for Health Research or the Department of Health and Social Care.

Competing interests: All authors have completed the ICMJE uniform disclosure form at www.icmje.org/coi disclosure.pdf, and declare: SJ, FA, JL, HB, RM, RR, HB are funded in full or in part by the National Institute for Health Research ARC North Thames. RR and NJF are NIHR Senior Investigators; no financial relationships with any organisations that might have an interest in the submitted work in the previous 3 years; no other relationships or activities that could appear to have influenced the submitted work.

Ethics approval and consent to participate: This study involved human participants and was reviewed and approved by NHS North West - Liverpool East Research Ethics Committee (16/NW/0654). Before taking part, all participants gave informed consent to participate in the study and for be analysed and reported anonymously.

Provenance and peer review: Not commissioned; externally peer reviewed.

Data availability statement: Due to the professional sensitivity of these data, qualitative data are not publicly available. Informed consent did not extend to use of these data outside the study.

Consent for publication: Not applicable

Availability of data and materials: Consent was not given by participants to share their anonymised transcripts.

For peer review only

ORCID iDs:

Sarah Jasim http://orcid.org/0000-0003-3940-6350

Fiona Aspinal http://orcid.org/0000-0003-3170-7570

Raj Mehta http://orcid.org/0000-0002-4003-530X

Jean Ledger http://orcid.org/0000-0003-2523-7971

Rosalind Raine http://orcid.org/0000-0003-0904-749X

Naomi J Fulop http://orcid.org/0000-0001-5306-6140

Helen Barratt http://orcid.org/0000-0002-1387-137X

For peer review only

For peer review only - http://bmjopen.bmj.com/site/about/guidelines.xhtml

REFERENCE LIST

1. National Institute of Clinical Excellence (NICE) Hip, knee and shoulder joint replacement guideline scope, 2018. Available from: https://www.nice.org.uk/guidance/gid-ng10084/documents/draft-scope Accessed Nov 18, 2021.
2. Briggs T. Getting it Right First Time: improving the quality of orthopaedic care within the National Health Service in England, 2012. London, United Kingdom: British Orthopaedic Association.
3. OECD. Hip and knee replacement. Health at a glance, 2019. OECD Indicators. Available from: https://www.oecd-ilibrary.org/sites/2fc83b9a-en/index.html?itemId=/content/component/2fc83b9a-en Accessed Nov 18, 2021.
4. Marsh M, Newman S. Trends and developments in hip and knee arthroplasty technology. *J Rehabil Assist Technol Eng*. 2021;8:2055668320952043. Published 2021 Feb 8. doi:10.1177/2055668320952043
5. Abdelaal MS, Restrepo C, Sharkey PF. Global Perspectives on Arthroplasty of Hip and Knee Joints. *Orthop Clin North Am.* 2020 Apr;51(2):169-176. doi: 10.1016/j.ocl.2019.11.003.
6. Carr A, Smith J A, Camaradou J et al. Growing backlog of planned surgery due to covid-19. BMJ 2021;372:n339. doi:10.1136/bmj.n339
7. Schrijvers G, van Hoorn A, Huiskes N. The care pathway: concepts and theories: an introduction. *Int J Integr Care.* 2012;12(Spec Ed Integrated Care Pathways):e192. Published 2012 Sep 18. doi:10.5334/ijic.812
8. Barbieri A, Vanhaecht K, Van HP et al. Effects of clinical pathways in the joint replacement: A meta-analysis. *BMC Med.* 2009;7:1–11. doi: 10.1186/1741-7015-7-32.
9. Centre for Policy on Ageing. Rapid review: The effectiveness of care pathways in health and social care, 2014. London, United Kingdom: CPA
10. Vanhaecht K, Panella M, van Zelm R et al. An overview on the history and concept of care pathways as complex interventions. *Int. J. Care Pathw.* 2010;14:117–123. doi: 10.1258/jicp.2010.010019.
11. Vanhaecht K. Ph.D. Dissertation. The Katholieke Universiteit Leuven (KU Leuven); Leuven, Belgium: 2007. The Impact of Clinical Pathways on the Organisation of Care Processes.
12. Teisberg E, Wallace S, O'Hara S. Defining and implementing value-based health care: A strategic framework. *Acad. Med*. 2020;95:682–685. doi: 10.1097/ACM.0000000000003122.
13. Berwick DM, Nolan TW, Whittington J. The triple aim: Care, health, and cost. *Health Aff.* 2008;27:759–769. doi: 10.1377/hlthaff.27.3.759.
14. Seys D, Bruyneel L, Deneckere S et al. Better organized care via care pathways: A multicenter study. PLoS ONE. 2017;12:1–11. doi: 10.1371/journal.pone.0180398.
15. Tremblay S-GA, Devitt KS, Kagedan DJ et al. The impact of a clinical pathway on patient postoperative recovery following pancreaticoduodenectomy. HPB (Oxford). 2017;19:799–807. doi: 10.1016/j.hpb.2017.04.015
16. Barratt, H, Turner, S, Hutchings, A, et al. Mixed methods evaluation of the Getting it Right First Time programme - improvements to NHS orthopaedic care in England: study protocol. *BMC Health Serv Res.* 2017 Jan 23;17(1):71. doi: 10.1186/s12913-017-2012-y. PMID: 28115018; PMCID: PMC5260012.
17. Getting It Right First Time. Effective Hip and Knee Replacement Pathway, 2020. Available from: https://gettingitrightfirsttime.co.uk/wp-content/uploads/2020/08/GIRFT-Hip-and-Knee-replacement-pathway-May-2020-003.pdf Accessed April 29, 2021.

For peer review only - http://bmjopen.bmj.com/site/about/guidelines.xhtml

18. Van Citters AD, Fahlman C, Goldmann DA, et al. Developing a pathway for high-value, patient-centered total joint arthroplasty. *Clin Orthop Relat Res.* 2014;472(5):1619-1635. doi:10.1007/s11999-013-3398-4
19. Goldsmith LJ, Suryaprakash N, Randall E et al. The importance of informational, clinical, and personal support in patient experience with total knee replacement: a qualitative investigation. *BMC Musculoskelet Disord.* 2017 Dec 24; 18 (1):127. doi: 10.1186/s12891-017-1474-8.
20. Conner-Spady BL, Marshall DA, Hawker GA, et al. You'll know when you're ready: a qualitative study exploring how patients decide when the time is right for joint replacement surgery. *BMC Health Serv Res*. 2014; 14:454. doi: 10.1186/1472-6963-14-454.
21. Sjöling M, Norbergh K-G, Malker H et al. What information do patients waiting for and undergoing arthroplastic surgery want? Their side of the story. *J Orthop Nurs*. 2006;10:5–14
22. Webster F, Perruccio AV, Jenkinson R et al. Where is the patient in models of patient-centred care: a grounded theory study of total joint replacement patients. *BMC Health Serv Res.* 2013 Dec 23;13:531. doi: 10.1186/1472-6963-13-531.
23. Carr T, Teucher UC, Casson AG. Time while waiting: patients' experiences of scheduled surgery. Qual Health Res. 2014 Dec;24(12):1673-85. doi: 10.1177/1049732314549022. Epub 2014 Sep 5.
24. Jeffery AE, Wylde V, Blom AW et al. 'It's there and I'm stuck with it': Patients' experiences of chronic pain following total knee replacement surgery. *Arthritis Care Res.* 2011;63:286–92
25. Doyle C, Lennox L, Bell DA. Systematic review of evidence on the links between patient experience and clinical safety and effectiveness. *BMJ Open* 2013;3:e001570. doi: 10.1136/bmjopen-2012-001570
26. Bate P, Robert G. Experience-based design: from redesigning the system around the patient to co-designing services with the patient. *BMJ Quality & Safety* 2006;15:307-310.
27. Barratt H, Hutchings A, Pizzo E et al. Mixed methods evaluation of the Getting it Right First Time programme in elective orthopaedic surgery in England: an analysis from the National Joint Registry and Hospital Episode Statistics. *BMJ Open* 2022;0:e058316. doi:10.1136/bmjopen-2021-058316.
28. Kitzinger J. Qualitative research: Introducing focus groups. BMJ 1995;311:299
29. Focus Group: A Practical Guide for Applied Research. 5th Edition. Richard A. Krueger and Mary Anne Casey. 2015. Sage Publishing ISBN: 978-1-4833-6524-4.
30. Ledger J, Mehta R, Jasim S et al. O12 Democratising the research process: reflections on the co-production of focus groups. *BMJ Open*, 2019, *9*(Suppl 1), A5–A5. https://doi.org/10.1136/bmjopen-2019-qhrn.12
31. NHS England. Transforming musculoskeletal and orthopaedic elective care services: rethinking referrals (NHSE), 2017. Available from: https://www.england.nhs.uk/wp-content/uploads/2017/11/msk-orthopaedic-elective-care-handbook-v2.pdf Accessed 1 May 2020
32. Brod M, Tesler LE, Christensen TL. Qualitative research and content validity: developing best practices based on science and experience. *Qual Life Res* 2009; 18: 1263–78.
33. Gale NK, Heath G, Cameron E et al. Using the framework method for the analysis of qualitative data in multi-disciplinary health research. *BMC Med Res Methodol* 2013, 13, 117. https://doi.org/10.1186/1471-2288-13-117
34. Mota REM, Tarricone R, Ciani, O et al. Determinants of demand for total hip and knee arthroplasty: a systematic literature review. BMC Health Serv Res,2012. 12, 225. https://doi.org/10.1186/1472-6963-12-225

For peer review only - http://bmjopen.bmj.com/site/about/guidelines.xhtml

35. Grocott MP. Pathway redesign: putting patients ahead of professionals. Clin Med (Lond). 2019;19(6):468-472. doi:10.7861/clinmed.2019-0292

For peer review only

For peer review only - http://bmjopen.bmj.com/site/about/guidelines.xhtml

Figure 1. Eligibility criteria

Figure 2. Patient pathway diagram

For peer review only

For peer review only - http://bmjopen.bmj.com/site/about/guidelines.xhtml

Figure 1: Eligibility criteria

All groups:

- Understand and speak English well enough to be able to give informed consent and participate in a group discussion
- Cognitively able to give informed consent and participate in a group discussion
- Not on a waiting list for total joint arthroplasty (TJA) (in case discussions affect their willingness to undertake TJA surgery)
- Aged over 18 years

Patient groups:

- Undergone elective TKA or THA between March 2016 and March 2018 (to minimise recall bias)
- Received **only** unplanned THA or TKA (because this is a different pathway)

Public group:

- Aged 60 years or older
- have **not** had TJA, but are willing to discuss NHS services to treat joint problems from a public perspective

For peer review only

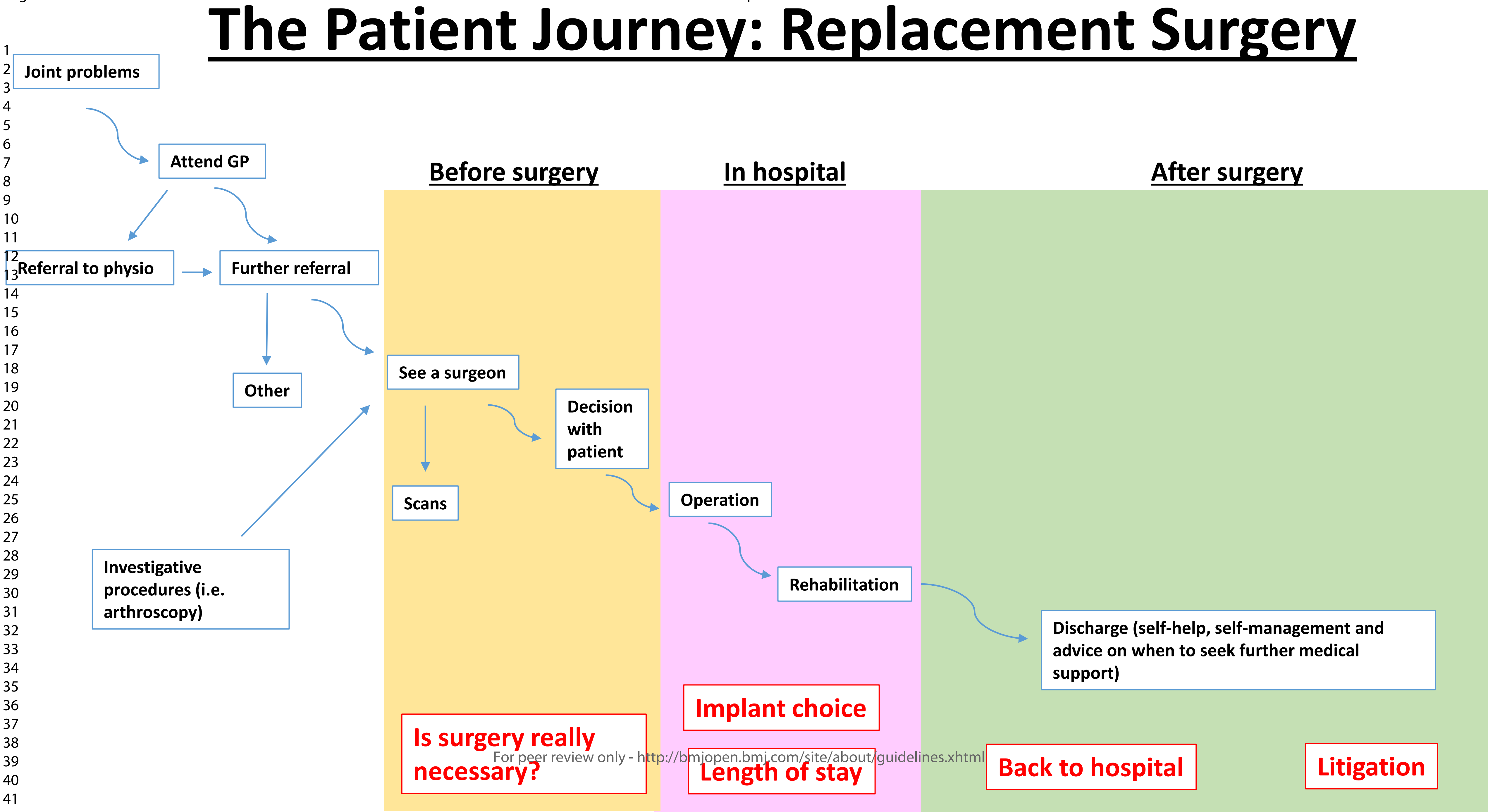

For peer review only - http://bmjopen.bmj.com/site/about/guidelines.xhtml

SUPPLEMENTAL MATERIAL

Online Supplementary File 1: SUMMARY TOPIC GUIDE 1 – GROUPS 1 & 2 (PATIENTS)

SECTION 1: EXPLORING PRACTICE: BEFORE, DURING AND AFTER SURGERY

Looking at this picture of the patient journey, we want to find out what was important to you at different stages:

1. **Before surgery** (orange box):

   At the point at which you were deciding whether to have a hip / knee replacement operation, what was important for you to know about?

2. **In hospital** (pink box):

   Based on your experience of having planned joint surgery in the NHS:

3. **After surgery** (green box):

   Once at home and following surgery, did you need to contact the NHS hospital where you were treated for any reason?

SECTION 2: EXPLORING GIRFT PROGRAMME PRIORITIES

4. In the presentation, we talked about how the GIRFT programme is trying to help hospitals across England provide similar 'results' for all patients who undergo planned orthopaedic surgery (e.g. to improve their life and reduce pain).

5. Are there any GIRFT programme priorities that were covered in the presentation today that we have not discussed and are also important to you?

6. Is there anything else important to you about joint surgery in the NHS, different from the GIRFT programme priorities and that we haven't talked about today?

SECTION 3: CLOSING COMMENTS

7. Finally, if you could give some feedback to the people leading this programme – what might you say?

For peer review only - http://bmjopen.bmj.com/site/about/guidelines.xhtml

Online Supplementary File 2: SUMMARY TOPIC GUIDE 2 – GROUP 3 (PUBLIC INDIVIDUALS)

SECTION 1: EXPLORING PRACTICE: BEFORE, DURING AND AFTER SURGERY

Looking at this picture of the patient journey, we want to find out what is important to you at different stages:

1. **Before surgery** (orange box):

   Scenario: Your GP has recommended that you see an orthopaedic surgeon, as you might need joint replacement surgery (e.g. a hip or knee replacement)

2. **After surgery** (green box):

   Scenario: Imagine you are at home after your hip/ knee replacement operation, and you become concerned about your recovery:

SECTION 2: EXPLORING GIRFT PROGRAMME PRIORITIES

3. In the presentation, we talked about how the GIRFT programme is trying to help hospitals across England provide similar 'results' for all patients who undergo planned orthopaedic surgery (e.g. to improve their life and reduce pain).

4. Are there any GIRFT programme priorities that were covered in the presentation today that we have not discussed and are also important to you?

5. Is there anything else important to you about joint surgery in the NHS, different from the GIRFT programme priorities and that we haven't talked about today?

SECTION 3: CLOSING COMMENTS

6. Finally, if you could give some feedback to the people leading this programme – what might you say?

For peer review only

Online Supplementary File 3: DETAILED STATEMENT FOR REFLEXIVITY

Reflexivity relates to acknowledgment of sensitivity to the ways in which the researchers and the research process may have shaped the data collected, including the role of prior assumptions and experience.[1]

Wider context of the research

Each focus group began with a short presentation explaining the context of the wider 'Getting It Right First Time' (GIRFT) [2] programme in elective orthopaedic surgery in England, and the programme priorities, to ensure all participants had a minimum level of knowledge. During analysis and interpretation, this pre-information was considered, and the possibility of how this knowledge might have shaped what was said.

Professional background of the research team

Members of the research team involved in fieldwork considered the ways in which their interactions with participants might be influenced by their own professional background, experiences, and prior assumptions. Co-facilitators (SJ and JL) were both academic research fellows from non-clinical backgrounds, the patient advisor (RM) who also co-facilitated focus groups had a non-clinical background but had previous experience of non-orthopaedic surgery. The observers (FA and HB) were both senior academic researchers who had clinical backgrounds. During our analysis and interpretation stages we considered whether knowing about our professional backgrounds could have impacted on participants' willingness to openly talk and share their experiences.

Potential for harm or distress

Members of the research team involved in fieldwork were sensitive to the possibility that focusing on the research topic (i.e. recalling previous surgical / in-hospital experiences) could potentially provoke anxiety in the focus group participants concerning the disclosure of adverse events. At the start of each focus group, co-facilitators (RM, SJ, JL) ensured that focus group participants could step out at any time or reveal or disclose as much of their previous experiences and perspectives as they felt comfortable with. At the end of each focus group, the research team stayed behind in the room to ensure that participants had the opportunity to seek support if they were feeling distressed by their participation. No focus group participants expressed such concerns or appeared to be distressed or uneasy.

Collaboration in knowledge production

Collaborative research is valued for its ability to bring together multiple researchers with distinctive and specialist perspectives to tackle large or complex research problems. There was a strong commitment within the research team from the outset to work collaboratively in the collection,

For peer review only - http://bmjopen.bmj.com/site/about/guidelines.xhtml

analysis, interpretation and reporting of the qualitative data, though individual involvement with the various stages of the research process varied as necessary. Team members closely involved in fieldwork (SJ, JL, RM, FA, HB) met frequently on separate occasions to discuss the progress of fieldwork and reflect on data collection. Meetings intensified during the initial stages of data collection and early analysis, when the topic guides and focus group materials were being produced and piloted, and themes and codes were beginning to be identified. At these crucial stages, input was sought from a senior member of the research team with extensive experience of qualitative research (NJF). In the final stages of analysis, input was sought from another senior member of the research team to sense check the coherence, truthfulness, and accuracy of the findings (RR). This resulted in an analytic strategy that was informed by insights from team members with a broad understanding of the research field and methodological issues, and those with field-based contextual and experiential understanding.

For peer review only - http://bmjopen.bmj.com/site/about/guidelines.xhtml

REFERENCE LIST

1. Jootun D, McGhee G, Marland G R. Reflexivity: promoting rigour in qualitative research. *Nurs stand.* 2009;23(23): 42-47.
2. Briggs T. Getting it Right First Time: improving the quality of orthopaedic care within the National Health Service in England, 2012. London, United Kingdom: British Orthopaedic Association.

For peer review only

Standards for Reporting Qualitative Research (SRQR)*

http://www.equator-network.org/reporting-guidelines/srqr/

Title and abstract

	Page/line no(s).
**Title** - Concise description of the nature and topic of the study Identifying the study as qualitative or indicating the approach (e.g., ethnography, grounded theory) or data collection methods (e.g., interview, focus group) is recommended	1
**Abstract** - Summary of key elements of the study using the abstract format of the intended publication; typically includes background, purpose, methods, results, and conclusions	2

Introduction

**Problem formulation** - Description and significance of the problem/phenomenon studied; review of relevant theory and empirical work; problem statement	4
**Purpose or research question** - Purpose of the study and specific objectives or questions	4

Methods

**Qualitative approach and research paradigm** - Qualitative approach (e.g., ethnography, grounded theory, case study, phenomenology, narrative research) and guiding theory if appropriate; identifying the research paradigm (e.g., postpositivist, constructivist/ interpretivist) is also recommended; rationale**	5
**Researcher characteristics and reflexivity** - Researchers' characteristics that may influence the research, including personal attributes, qualifications/experience, relationship with participants, assumptions, and/or presuppositions; potential or actual interaction between researchers' characteristics and the research questions, approach, methods, results, and/or transferability	6 & Supplement
**Context** - Setting/site and salient contextual factors; rationale**	6
**Sampling strategy** - How and why research participants, documents, or events were selected; criteria for deciding when no further sampling was necessary (e.g., sampling saturation); rationale**	5
**Ethical issues pertaining to human subjects** - Documentation of approval by an appropriate ethics review board and participant consent, or explanation for lack thereof; other confidentiality and data security issues	6
**Data collection methods** - Types of data collected; details of data collection procedures including (as appropriate) start and stop dates of data collection and analysis, iterative process, triangulation of sources/methods, and modification of procedures in response to evolving study findings; rationale**	6

For peer review only

**Data collection instruments and technologies** - Description of instruments (e.g., interview guides, questionnaires) and devices (e.g., audio recorders) used for data collection; if/how the instrument(s) changed over the course of the study	6
**Units of study** - Number and relevant characteristics of participants, documents, or events included in the study; level of participation (could be reported in results)	5
**Data processing** - Methods for processing data prior to and during analysis, including transcription, data entry, data management and security, verification of data integrity, data coding, and anonymization/de-identification of excerpts	6
**Data analysis** - Process by which inferences, themes, etc., were identified and developed, including the researchers involved in data analysis; usually references a specific paradigm or approach; rationale**	6
**Techniques to enhance trustworthiness** - Techniques to enhance trustworthiness and credibility of data analysis (e.g., member checking, audit trail, triangulation); rationale**	6

Results/findings

**Synthesis and interpretation** - Main findings (e.g., interpretations, inferences, and themes); might include development of a theory or model, or integration with prior research or theory	7-12
**Links to empirical data** - Evidence (e.g., quotes, field notes, text excerpts, photographs) to substantiate analytic findings	7-12

Discussion

**Integration with prior work, implications, transferability, and contribution(s) to the field** - Short summary of main findings; explanation of how findings and conclusions connect to, support, elaborate on, or challenge conclusions of earlier scholarship; discussion of scope of application/generalizability; identification of unique contribution(s) to scholarship in a discipline or field	13
**Limitations** - Trustworthiness and limitations of findings	3 & 13

Other

**Conflicts of interest** - Potential sources of influence or perceived influence on study conduct and conclusions; how these were managed	14
**Funding** - Sources of funding and other support; role of funders in data collection, interpretation, and reporting	14

*The authors created the SRQR by searching the literature to identify guidelines, reporting standards, and critical appraisal criteria for qualitative research; reviewing the reference lists of retrieved sources; and contacting experts to gain feedback. The SRQR aims to improve the transparency of all aspects of qualitative research by providing clear standards for reporting qualitative research.

For peer review only

For peer review only - http://bmjopen.bmj.com/site/about/guidelines.xhtml

**The rationale should briefly discuss the justification for choosing that theory, approach, method, or technique rather than other options available, the assumptions and limitations implicit in those choices, and how those choices influence study conclusions and transferability. As appropriate, the rationale for several items might be discussed together.

Reference:
O'Brien BC, Harris IB, Beckman TJ, Reed DA, Cook DA. **Standards for reporting qualitative research: a synthesis of recommendations.** *Academic Medicine*, Vol. 89, No. 9 / Sept 2014 DOI: 10.1097/ACM.0000000000000388

For peer review only

BMJ Open

‘Maze’ not pathway: focus group exploration of patients’ and public experiences of the UK NHS elective total joint arthroplasty pathway.

Journal:	*BMJ Open*
Manuscript ID	bmjopen-2022-066762.R1
Article Type:	Original research
Date Submitted by the Author:	12-Jun-2023
Complete List of Authors:	Jasim, Sarah; The London School of Economics and Political Science, Care Policy and Evaluation Centre<br>Aspinal, Fiona; University College London, Department of Applied Health Research<br>Mehta, Raj; University College London, Department of Applied Health Research<br>Ledger, Jean; University College London<br>Raine, Rosalind; University College London, Institute of Epidemiology & Health<br>Fulop, Naomi; University College London, Applied Health Research<br>Barratt, Helen; UCL, Department of Applied Health Research
**Primary Subject Heading**:	Health services research
Secondary Subject Heading:	Qualitative research
Keywords:	Orthopaedic & trauma surgery < SURGERY, QUALITATIVE RESEARCH, Hip < ORTHOPAEDIC & TRAUMA SURGERY, Knee < ORTHOPAEDIC & TRAUMA SURGERY, Quality in health care < HEALTH SERVICES ADMINISTRATION & MANAGEMENT

SCHOLARONE™
Manuscripts

I, the Submitting Author has the right to grant and does grant on behalf of all authors of the Work (as defined in the below author licence), an exclusive licence and/or a non-exclusive licence for contributions from authors who are: i) UK Crown employees; ii) where BMJ has agreed a CC-BY licence shall apply, and/or iii) in accordance with the terms applicable for US Federal Government officers or employees acting as part of their official duties; on a worldwide, perpetual, irrevocable, royalty-free basis to BMJ Publishing Group Ltd ("BMJ") its licensees and where the relevant Journal is co-owned by BMJ to the co-owners of the Journal, to publish the Work in this journal and any other BMJ products and to exploit all rights, as set out in our licence.

The Submitting Author accepts and understands that any supply made under these terms is made by BMJ to the Submitting Author unless you are acting as an employee on behalf of your employer or a postgraduate student of an affiliated institution which is paying any applicable article publishing charge ("APC") for Open Access articles. Where the Submitting Author wishes to make the Work available on an Open Access basis (and intends to pay the relevant APC), the terms of reuse of such Open Access shall be governed by a Creative Commons licence – details of these licences and which Creative Commons licence will apply to this Work are set out in our licence referred to above.

Other than as permitted in any relevant BMJ Author's Self Archiving Policies, I confirm this Work has not been accepted for publication elsewhere, is not being considered for publication elsewhere and does not duplicate material already published. I confirm all authors consent to publication of this Work and authorise the granting of this licence.

For peer review only

'Maze' not pathway: focus group exploration of patients' and public experiences of the UK NHS elective total joint arthroplasty pathway.

*Sarah Jasim, Care Policy and Evaluation Centre, London School of Economics and Political Science, Houghton Street, Holborn, London, WC2A 2AE, UK s.jasim@lse.ac.uk

Sarah Jasim, Care Policy and Evaluation Centre, London School of Economics and Political Science, UK

Fiona Aspinal, Department of Applied Health Research, University College London, UK

Raj Mehta, Patient Advisor, NIHR ARC North Thames Research Advisory Panel, Department of Applied Health Research, University College London, UK

Jean Ledger, Department of Applied Health Research, University College London, UK

Rosalind Raine, Department of Applied Health Research, University College London, UK

Naomi J. Fulop, Department of Applied Health Research, University College London, UK

Helen Barratt, Department of Applied Health Research, University College London, UK

*corresponding author

Word count: 3189 words

For peer review only

For peer review only - http://bmjopen.bmj.com/site/about/guidelines.xhtml

ABSTRACT

Objective: To explore patient and public perceptions of planned improvements to the NHS TJA pathway.

Design: Three qualitative focus groups undertaken March – May 2018, as part of a mixed-methods evaluation of GIRFT. Transcripts were subject to framework analysis to identify thematic content between October 2018 – October 2021.

Setting: Elective TJA surgery in the English NHS.

Participants: Two focus groups including patients who had undergone TJA in the previous two years (Group 1: n=5; Group 2: n=4) and the other individuals who had not but were aged 60+ (Group 3: n= 5). Participants were recruited via community groups and patient panels.

Results: Fourteen individuals took part in the focus groups; all were aged over 60; seven (50%) were female; and nine (64%) had undergone TJA surgery. Participants' perspectives were categorised into themes and mapped onto stages of the TJA pathway. Although perioperative care is often the focus of improvement efforts, participants argued that the patient journey begins before individuals present to primary care. Participants had concerns about other aspects of the pathway, such as obtaining a surgical referral, with pre-referral interventions aimed at potentially avoiding the need for surgery (i.e. physiotherapy) being perceived as a mechanism to restrict access to secondary care. Patient experience was also conceptualised as a 'maze', rather than the logical, sequential process set out in clinical guidelines; exacerbated by a lack of information, knowledge, and power imbalances.

Conclusion: The linear conceptualisation of the TJA pathway is at odds with patient experience. Improvement programmes focussed on perioperative care fail to consider patient concerns and priorities. Patients should be directly involved in improvement programmes, to ensure that patient experience is optimised, as well as informing related processes and important outcomes of care.

For peer review only

STRENGTHS AND LIMITATIONS OF THIS STUDY

- This is the first study to look at the entire TJA pathway from the perspective of patients and the public and explore how their priorities differ from those of clinicians and managers seeking to improve care.
- Co-producing research with a patient advisor ensured the patient perspective was central to our work from planning through to analysis and dissemination.
- We sought to recruit via a wide range of different channels, including professional societies, patient groups, and local community groups (including multi-faith organisations).
- Nevertheless, we were not able to recruit a diverse sample with respect to ethnicity, and one participant withdrew because of fears of cultural stigma.
- As participants volunteered to participate, there may be a degree of self-selection bias, with participants who were more confident in articulating their views and experiences or indeed, those with particular types of experiences, potentially more likely to participate.

For peer review only

BACKGROUND

Elective hip and knee replacement are the two most common total joint arthroplasty (TJA) procedures, and two of the most frequently performed and effective surgical procedures in the UK [1,2] and globally.[3] With an increasingly ageing population, and trend for TJA earlier in life,[4] the predicted growth in global demand is likely to have a significant impact on health systems,[5] such as the National Health Service (NHS) in England.

A care pathway [6] is a multidisciplinary tool for planning the care of specific, well-defined groups of patients who have a predictable clinical course, such as TJA. Drawing on research evidence and best practice, it sets out how different components of care should be optimised and sequenced, to enhance quality [7] and improve patient safety. This approach has been shown to reduce unnecessary variation [6,8-11] and may improve patient outcomes and optimise resource use.[6,12-14] Standardisation of care is important for national improvement programmes such as the English 'Getting It Right First Time' programme (GIRFT), which aims to tackle unwarranted variation in orthopaedic surgery across the NHS.[15,16] GIRFT sought to develop a 'best of the best' clinical pathway [17] to improve patient experience and expedite recovery following TJA. Although clinical perspectives are central to pathway development,[8] the views of current and future patients who must navigate them, are equally important to optimise experience, outcomes, and efficiency.[18]

In the UK, general practitioners (GPs) act as gatekeepers to secondary care, including the TJA pathway. [19] Patients experiencing hip or knee pain typically first present to a GP. In some English regions, local commissioners (leaders responsible for purchasing health care on behalf of local communities) are increasingly setting criteria that patients must meet before they can be referred to an orthopaedic surgeon. Current guidance recommends conservative management initially, for at least three months, with the aim of potentially avoiding the need for the patient to undergo surgery. Interventions include medication, physiotherapy, and/or support with lifestyle and weight loss. If this is not successful, the GP should refer the patient to an orthopaedic surgeon to discuss potential surgery.

Existing research about patient perspectives of the TJA pathway has tended to focus on patient satisfaction and experience [20] at specific stages of the pathway, such as deciding to undergo surgery,[21] pre-operative education,[22] waiting for surgery,[23-24] and management of post-operative pain.[12] Given shared decision-making is considered integral to care pathways as well as wider efforts to improve care, there is currently no evidence on how patients and public view planned improvements to the TJA pathway, and whether their priorities align with those of clinicians and managers.[25,26] This study sought to address that gap by exploring patient and public perceptions of planned improvements to the TJA pathway, including the factors that influenced this. The research was part of a wider mixed methods evaluation of the GIRFT programme.[15,16,27]

For peer review only - http://bmjopen.bmj.com/site/about/guidelines.xhtml

METHODS

Study Design and Recruitment

Focus groups enabled us to collect views from multiple participants simultaneously and explore meanings behind those views, which were generated, discussed, and refined through group interactions in a way that could not happen with other methods.[28]

To explore the outcome of interest, which was patient and public perceptions of planned improvements to the NHS TJA pathway, we sought to recruit between four to eight participants to each of three focus groups. This was based on evidence regarding the optimal number of groups and participants for focus group research.[29] Groups 1 and 2 included patients who had undergone an elective hip or knee replacement in the previous two years (March 2016–March 2018). Group 3 included individuals who had not undergone TJA but were in the age bracket most likely to receive this type of intervention (age 60+) (Figure 1).[30] This enabled us to compare experiences of those who had undergone TJA with the expectations of those who had not, and how this influenced participants' views. In this paper we report the findings from all three groups together, referring to individuals as participants, except where there were clear differences between those who had undergone surgery, and those who had not. We recruited participants via community organisations across London (UK) whose membership was likely to be aged 60 and over (e.g. third sector organisations, day centres and activity clubs). We also invited participation from eligible patient advisory group members from relevant organisations (e.g. the British Orthopaedic Association). Researchers (SJ, JL) conducted telephone and e-mail screening and collected preliminary demographic information to maximise sample diversity (Table 1).

Table 1: Participant characteristics

	**Group 1**<br>(Patients – n=5)	**Group 2**<br>(Patients – n=4)	**Group 3**<br>(Public – n=5)
**Sex**	Male (n=2), Female (n=3)	Male (n=2), Female (n=2)	Male (n=3), Female (n=2)
**Age range**	60-69 (n=2)<br>70-79 (n=3)	60-69 (n=1)<br>70-79 (n=1)<br>80-89 (n=2)	60-69 (n=3)<br>70-79 (n=2)
**Ethnicity**	White British (n=4)<br>White European (n=1)	White British (n=3)<br>White South African (n=1)	White British (n=4)<br>White North American (n=1)

For peer review only - http://bmjopen.bmj.com/site/about/guidelines.xhtml

Data Collection

We conducted three focus groups between March to May 2018 in an academic (non-healthcare) setting. Observation data were collected by senior researchers (FA or HB) to capture any contextual factors that might have impacted data quality, such as the topics being discussed or depth of discussion. All participants gave informed consent. Each focus group began with a brief presentation by the research team, providing a high-level overview of efforts to improve the TJA pathway for patients, including GIRFT.[15]

Focus group discussions were informed by semi-structured topic guides (online supplementary file 1-2), which included participants' priorities before, during and after surgery, as well as their views of the planned improvements to the TJA pathway. Each group lasted approximately two hours and was audio-recorded using an encrypted device for professional transcription in full.

Patient and Public Involvement

Three focus groups were co-facilitated by a researcher (SJ or JL) and the patient advisor (RM).[31] Following advice from our patient advisor (RM),[31] we (SJ) also provided participants with a simplified illustration of the UK TJA care pathway (Figure 2), based on clinical guidance at the time.[32] The pathway diagram and topic guides were piloted with a research advisory panel of patient advisers and refined initially, and then iteratively as the research progressed (i.e., diagram title and emphasis of questions), to take account of emerging findings, in line with accepted qualitative methods.[33] Preliminary results of the study were verbally presented to participating patient advisors.

Data Analysis

The core research team (HB, FA, RM, SJ, JL) held de-brief meetings after each focus group to ensure triangulation between focus group findings and researcher observations. Observation notes and field notes were not subject to formal analysis but were used in debrief and analysis meetings to ensure transcript data were contextualised. Transcript data were analysed using a Framework approach.[34] We used the care pathway diagram (Figure 2) and structure of the topic guides (online supplementary file 1-2) to develop the initial coding framework, and incorporated concepts and meanings identified in the data.[13]

The first stage of analysis involved familiarisation, where researchers (SJ, JL, FA, HB) read and collaboratively reviewed transcripts to understand meanings, concepts, and ideas, in a reflexive manner (see online supplementary file 3). The wider research team (SJ, JL, RM, FA, HB, NJF) then identified and confirmed parameters of the main themes to form the basis of a thematic framework in a series of separate meetings. Data were indexed based on the thematic framework using MAXQDA software (SJ). Indexed data were organised into thematic charts for each category, in Microsoft Excel, using direct quotations where possible. These were reviewed and modified when necessary to clarify meaning (FA). Themes were further mapped and interpreted to understand areas of significance to patients and the public (SJ, FA, HB, JL, RM, NJF, RR).

Ethics

Ethical approval was granted by NHS North West – Liverpool East Research Ethics Committee (16/NW/0654).[15]

For peer review only - http://bmjopen.bmj.com/site/about/guidelines.xhtml

RESULTS

Participants

Seventeen individuals agreed to participate, although three withdrew on the day. Two because of heavy snowfall (Group 2 and Group 3) and one due to fears of cultural stigma from openly discussing experiences (Group 2). All 14 participants were aged over 60 and all self-identified as White British or White Other. Seven were female, and 9 participants had undergone TJA surgery in the previous two years. Of the 5 who had not undergone surgery (i.e. Group 3), 3 had friends or family who had previously undergone TJA.

Our original goal was to explore participants' views about planned improvements to the TJA pathway (such as GIRFT),[2,15] which were focused particularly on the perioperative 'in-hospital' (Figure 2) phase of the care pathway. However, it quickly became apparent that other phases of the pathway were of equal, if not more, importance to participants. Participants understood the pathway diagram, but argued that whilst the streamlined, linear presentation may be how it appears to clinicians and managers, it did not reflect their experiences. Here, we report participants' views about priorities for pathway improvement; the realities of traversing the TJA pathway; and barriers and facilitators they encountered whilst navigating the pathway (participant quotations are provided in Table 2).

For peer review only

For peer review only - http://bmjopen.bmj.com/site/about/guidelines.xhtml

Table 2: Focus group (FG) quotes

Ref.	FG No.	Gender	Quote
Quote1	1	Male	'…… that's [pointing to the first part of the pathway diagram] probably where most problems, as far as I'm concerned, arose. In that trying to see a consultant was very difficult.'
Quote2	1	Male	'…I get a letter in the post saying, it's a confirmation of the date, so that would be followed by another letter that says they've just cancelled that appointment…With no explanation, it just says it's cancelled… "What happens, where are we now, or what happens now?"…they said, "Oh well, it's got to go in before the assessment committee"… Well, this is very, very … upsetting that… I'm in a lot of pain, I want to see a consultant, and there's all these obstacles in the way before you can even see the consultant.'
Quote3	1	Female	'I had months of physiotherapy right through till February the following year. It wasn't helping, I didn't think it was helping a lot... And then in the February I spoke to my physiotherapist, asked him… Am I going to go on and on like this? … So, I'm now thinking, "…they're just… shuffling me along," obviously … would I like to just go ahead with the op…'
Quote4	1	Male	'Yeah, I think it's a postcode lottery, isn't it, where some people live that there's a huge waiting list and I think the GPs…refer them to the physiotherapist first so… keeps them out of the way for a bit…'
Quote5	2	Male	'…the GP is the stopping point, and it causes a lot of the other things going up the line to be worse than they need to be because they resist. Obviously, they're the Clinical Commissioning Group that actually drives them on this and say, 'Don't refer, don't refer, don't refer, we'll even give you some extra bonus points if you don't refer.'
Quote6	2	Male	'…so it was down to you to look after your own, and when it all starts to go wrong, you've got to go to your GP and you've got to persuade him to refer you to a specialist…'
Quote7	1	Female	'…I felt I had to bully them in order to have the surgery…'
Quote8	1	Female	'– I was just taken aback by how much I felt like a widget on a production line.'
Quote9	3	Female	'…if you like, you'd need a referral to Social Services. I think that should be part of the pre-operative planning rather than suddenly realising afterwards. What I was talking about was the OT assessment and the follow-up physiotherapy, I wasn't talking about Social Services, about social care support, which you may well need and that should have really been part of the planning beforehand. Because if you've got to wait 'til afterwards you'll be in hospital for a month before anything happens. I know because I've been a social worker so, you know, I know what it's like.'
Quote10	1	Female	'lack of communication between various people, the doctors, the nurses, so that I felt I was the person who knew more than anyone else about what was going on, but I knew least about what should be going on'
Quote11	3	Female	'I'd like to know about physiotherapy and after care and the system they use…So I'd want to know, if I was going to go in, whether I'd be not only would the surgery be good and efficient but there would be a very good programme coming out in after care and who'd be doing that with me.'
Quote12	3	Female	'I want to come back to the Social Services and what happens when you get out of hospital, especially if you live alone or... you know, how do you manage for that period of time after hospital, that's an important handover and that's not always handled really well.'
Quote13	1	Male	'..like a maze'
Quote14	1	Male	'all the time there's never any communication as to what's happening. So, you're left in the dark, and that itself is upsetting because, you know, you want to get it dealt with, you don't know at what stage it's going to happen, and you're just being pushed from pillar to post, and nobody will give you any information.'
Quote15	3	Female	'There's a big thing about sort of communication through the line so that people don't rely on you as the rather disorientated patient after an operation to actually be the person who's informing so that there are other people sharing information, but you've also got a voice, that your voice is going to be heard or that you've elected someone to speak for you and that person's voice is going to be heard as well.'
Quote16	3	Male	'information tends to be... You're always given something when you go out - and this is all kinds of hospitalisation, not just surgery… but it's never quite enough… but it's generally not good…'

For peer review only - http://bmjopen.bmj.com/site/about/guidelines.xhtml

Quote17	1	Female	'because I can see from this chart [diagram] that I'm on a conveyor belt and that's the feeling that I had. And actually, I felt that I'd been in the dark from the start. And I felt that there is information there if I ask the right question, but I don't know what question to ask. And I have felt that I have to look it up a lot myself, I have to find out things myself'
Quote18	2	Female	"Well, personally I wasn't aware that there was even any choice."
Quote19	3	Female	'It is actually a plus point for hospitals to make their statistics available publicly in some way because people will search for excellence and people will avoid mediocrity, and I certainly don't depend on my GP for that...'
Quote20	1	Female	'So, that question of choice actually goes all the way through. Is it really choice or not and how much power do we really have? How much knowledge do we have?'
Quote21	1	Female	"I was so intimidated by him, and so overwhelmed by him, I thought, 'Right, okay, well, you know, I'm just going to … there's no point in my doing anything else here'."
Quote22	3	Female	'…care-givers are important too and I can't tell you how many times I was at a doctor and the doctor just ignored the questions that I had to ask because I was not the patient...'
Quote23	1	Female	'But it was about two weeks before my operation, so if you are anxious and you feel like you're in the dark, that's a long time to wait before you get those questions, a chance to answer those questions, and you find out new information. It was at the joint school that I found out that you don't have a general anaesthetic, that you have a spinal anaesthetic.'
Quote24	1	Female	'But then physio told me that there was a system whereby you get a choice of hospitals for having your operation in. I didn't know that either. She said, 'Haven't you heard about this before?' And she gave me a list of six hospitals, the physio… she said, 'Look them up, go and read the CQC report on these hospitals and see which one you like, and if you prefer one of the other ones, come back and let me know'. I'm sorry, having come through all this and been so in the dark I'm now being told to decide on what hospital.'
Quote25	1	Female	'I knew the system more, I was much more able to work my way through it, to talk to the right people, to make the decision'
Quote26	3	Female	'…I'd want the occupational therapist to assess me, assess my home, work out what the … support I'm going to get at home to actually help me achieve mobility and how accessible they are…'

Patient priorities: obtaining a referral to see an orthopaedic surgeon

Although the peri-operative phase of the pathway is often the focus of improvement programmes, such as GIRFT, individuals who had undergone TJA argued that obtaining a referral to an orthopaedic surgeon is in fact a greater challenge and concern (see Table 2, Quote1). This was 'upsetting' given their level of pain or disability (Quote2) especially when appointments were postponed. Some participants found that GP-recommended non-surgical interventions resulted in little or no improvement, whilst others had been in too much pain to engage with physiotherapy (Quote3). Consequently, they viewed this approach simply as a mechanism for managing demand (Quote4). Some participants believed that GPs had pressure placed on them to not refer patients to secondary care (Quote5,). Others felt that external pressures to ration NHS services impacted their access, such as referrals being reviewed by an 'assessment committee' (Quote2) or appointments being delayed by repeated, unexplained cancellations.

Because of the obstacles they had faced, participants spoke about the difficulties encountered to get onto the pathway and secure an orthopaedic referral, and in some cases, 'persuade' (Quote6-) or even 'bully' (Quote7-) healthcare professionals, whilst on this conveyor belt' (Quote17) or 'production line' (Quote8).

The realities of the elective TJA pathway

For peer review only - http://bmjopen.bmj.com/site/about/guidelines.xhtml

Participants, especially those who had not undergone surgery (Group 3), emphasised the importance of joined-up care planning throughout the pathway, including clinical teams planning surgery dates to coincide with available post-operative support (Quote9). Disconnected communication and ways of working between health providers at different stages of the pathway, left patients (Groups 1 and 2) having to assume responsibility for their own care, despite feeling powerless with insufficient information to make such informed decisions (Quote10). The lack of joined-up care was important to all groups and seen as being exacerbated by poor communication. For example, they described poor discharge procedures where communication with wider services was lacking, such as pharmacy, community health services, rehabilitative physiotherapy (Quote11) and social care (Quote12).

Navigating the 'maze'

Without joined-up forward-planning, patients had to manage and navigate a range of complex local health systems alone. They were left feeling adrift from the system, and many participants felt they were ill-equipped to tackle the obstacles they encountered because of asymmetries of both information and power. As one neatly summarised, the pathway is 'like a maze' (Quote13).

Lack of information and knowledge

Participants reported that from the point of referral from primary care, they were 'left in the dark' (Quote14). A lack of communication (Quote15) or appropriate, timely information from their healthcare provider hindered their understanding of the process (, , Quote16) making it difficult for them to make informed choices . This was compounded for some by a lack of knowledge, meaning that they felt they did not know what questions they should be asking. This was particularly a concern in the stages prior to surgery but differed according to participants' direct or indirect experiences; location of care; and pre-existing knowledge or professional background (Quote17).

Although clinical guidance [35] implies shared decision-making should be built into the pathway, participants' knowledge about available choices varied. For example, some participants in all groups had not known that they could choose the hospital they went to, or their surgeon, whilst others had sought out information to inform their choice (Quote18, Quote19). Nevertheless, even those who were aware that they could exercise choice, felt that they lacked sufficient knowledge to do so (Quote20).

Power imbalance

Alongside a lack of information and communication, many participants described how they had to disrupt existing power dynamics to navigate the TJA pathway. Asymmetries of power in relationships with key health professionals could significantly impact their experience of care (Quote8) and their progress along the pathway. For example, negative interactions with a surgeon had halted one participant's journey during their first appointment with a surgeon and hindered their access to pathway at the very start (Quote21). Another participant, who had cared for a TJA patient, described how her attempts to advocate for them were ignored (Quote22).

Overcoming obstacles

Participants described three distinct sources of knowledge which they drew on to differing degrees to overcome the obstacles they faced: formal learning; informal learning; and lived experience.

Formal learning

For peer review only - http://bmjopen.bmj.com/site/about/guidelines.xhtml

Formal learning opportunities offered to patients prior to surgery had helped some. For example, pre-operative education groups (also known as 'Joint Schools') run by hospital staff for patients listed for surgery, had alleviated fears, increased knowledge, and provided an opportunity to ask questions. Nevertheless, for some, the programme was offered very close to the date of the procedure, meaning that they had a long wait to raise their concerns. Consequently, some would have liked this to have been offered much earlier in the pathway (Quote23).

Informal learning

Some participants also sought out or expected to seek out information for themselves that they thought they needed to navigate the process. This included details about medication (carer, Group 3); chasing appointments (female patient, Group 1); and undertaking their own research to inform choices (all groups) and guide decision-making (female patient, Group 1). Others had also sought information from health professionals such as physiotherapists who were familiar with the TJA pathway, which helped them feel 'less in the dark' (Quote24).

Lived experience

Participants who were able to draw on a previous experience of undergoing surgery, used this to help inform choices and decision-making and felt better able to navigate the pathway on subsequent occasions. Those who had previously undergone a TJA knew to request additional information about regaining function, resuming activities, and exercising, not only to improve outcomes but also to assist navigating the crucial post-operative stage of the pathway (Quote26). Some also believed that this knowledge had enabled them to expedite the process on subsequent occasions, including identifying potential short cuts (Quote25,).

For peer review only

For peer review only - http://bmjopen.bmj.com/site/about/guidelines.xhtml

DISCUSSION

Despite ongoing efforts to streamline and standardise care, patient experience differs significantly from the seemingly logical, sequential process set out in clinical guidelines. [35] Importantly for patients, the pathway does not begin and end at the doors of the hospital. For example, it starts at first presentation to primary care with joint pain. A lack of timely information and communication also leaves patients feeling that they have been 'left in the dark' to navigate a 'maze'. This sense is exacerbated by power and information asymmetries between patients and clinicians. Whilst formal education programmes such as 'Joint School' can be helpful, many patients have to seek out additional information for themselves to answer their queries, understand the procedure and navigate the TJA pathway.

Previous research has largely focused on specific phases of the care pathway. This is the first study to look at the entire TJA pathway from the perspective of patients and the public and explore how their priorities differ from those of clinicians and managers seeking to improve care. Co-producing research with a patient advisor ensured the patient perspective was central to our work from planning through to analysis and dissemination. Qualitative data collection methods were used to allow participants to describe their experience and views in their own words, and the focus group approach enabled sharing, comparing and corroboration of ideas. As socio-economic circumstances may impact a patient's decision to undergo TJA,[36] we sought to recruit via a wide range of different channels, including professional societies, patient groups, and local community groups (including multi-faith organisations). Nevertheless we were not able to recruit a diverse sample with respect to ethnicity. Indeed, one participant withdrew because of fears of cultural stigma. It is acknowledged that cultural differences may exist between other groups of patient and public members. As participants volunteered to participate, there may be a degree of self-selection bias, with participants who were more confident in articulating their views and experiences or indeed, those with particular types of experiences, potentially more likely to participate. The methodology was selected to explore a range of views in depth, and not necessarily representation. The number of study participants may limit the transferability of findings – although this was mitigated using purposeful sampling there was geographical variability in participants' lived experiences of the UK TJA pathway. [37]

Our findings have several implications for clinicians and policymakers. Although perioperative care is often the focus of clinically led efforts (e.g. GIRFT) [2,27] to improve the TJA pathway, participants also emphasised that obtaining a referral to a surgeon is often the greatest challenge, and the post-operative period following surgery is a priority. Despite some evidence to suggest that prehabilitation is associated with improving some of the outcomes for patients undergoing orthopaedic surgery both preoperatively and postoperatively, we have highlighted that non-surgical interventions (e.g. physiotherapy) prior to surgical referral are perceived by patients and the public as a means of managing demand,. [38] While this remains the case, as well as potentially damaging patient trust, it is likely to impact patient engagement with evidence-based care. Efforts to provide patients with information about their procedure and likely recovery (e.g. via 'Joint Schools') are welcome, but many want information about the pathway earlier than this so that they are not left feeling ill-equipped to navigate the 'maze' of care themselves, especially if they have not previously undergone surgery. At a policy-level, those working to standardise services using care pathways should also be aware that patient experience is seldom analogous to their linear, stepwise conceptualisations. In line with the existing literature, our findings suggest that experience-based co-design should be a core part of pathway re-design.[39] Challenges with traversing the TJA pathway, alongside the negative impacts on physical and mental wellbeing of delayed appointments that were reported in this study, are likely to be exacerbated by the COVID-19 pandemic which has resulted in

For peer review only - http://bmjopen.bmj.com/site/about/guidelines.xhtml

further delays, cancellations, and increased waiting times for those on the TJA pathway. [40] The subsequent impacts on the health system have increased the length of patients' experience of pain, decreased mobility, worsened quality of life. [41,42]

CONCLUSION

In conclusion, we have highlighted the disparity between the priorities of improvement programmes such as GIRFT and those of patients and the public. Whilst improvement programmes typically seek to improve peri-operative care, patient concerns lie elsewhere, for example obtaining a referral to an orthopaedic surgeon and managing recovery at home. Research that includes views from both patients and health care professionals is needed at these crucial stages of the TJA pathway to explore how patients are being supported. The typically linear conceptualisation of TJA pathways in policy documents is also at odds with how patients experience the process. With rates of TJA set to increase globally, patients and public must be directly involved in improvement programmes to optimise not only processes and outcomes of care, but also patient experience.

For peer review only

For peer review only - http://bmjopen.bmj.com/site/about/guidelines.xhtml

DECLARATIONS

Acknowledgements: The authors would like to acknowledge and thank the focus group participants, and especially Steven Towndrow, Wendy Chandler, Stephanie Hume, Patricia Hallam and the NIHR ARC (formerly CLAHRC) North Thames Research Advisory Panel patient advisers for their efforts in preparation and organisation of the focus groups.

Author Contributions: HB was principal investigator. HB and RR initiated the research. HB, NJF and RR designed the evaluation. SJ, RM and JL were responsible for qualitative data collection; SJ, FA,RM, JL, NJF and HB analysed the qualitative data. SJ, FA, RM, JL, RR, NJF and HB drafted the manuscript and all authors contributed to reviewing and substantive revision. All authors approved the final version. All authors had full access to all the data in the study and accept responsibility to submit for publication. HB will act as guarantor.

Funding: National Institute for Health and Care Research (NIHR) Programme Grants for Applied Research (Applied Research Collaboration (ARC) North Thames)

Disclaimer: This report is independent research funded by the National Institute for Health Research ARC North Thames. The views expressed in this publication are those of the authors and not necessarily those of the National Institute for Health Research or the Department of Health and Social Care.

Competing interests: No competing interests.

Ethics approval and consent to participate: This study involved human participants and was reviewed and approved by NHS North West - Liverpool East Research Ethics Committee (16/NW/0654). Before taking part, all participants gave informed consent to participate in the study and for be analysed and reported anonymously.

Provenance and peer review: Not commissioned; externally peer reviewed.

Data availability statement: No data are available.

Consent for publication: Not applicable

Availability of data and materials: Consent was not given by participants to share their anonymised transcripts.

For peer review only

For peer review only - http://bmjopen.bmj.com/site/about/guidelines.xhtml

ORCID iDs:

Sarah Jasim http://orcid.org/0000-0003-3940-6350

Fiona Aspinal http://orcid.org/0000-0003-3170-7570

Raj Mehta http://orcid.org/0000-0002-4003-530X

Jean Ledger http://orcid.org/0000-0003-2523-7971

Rosalind Raine http://orcid.org/0000-0003-0904-749X

Naomi J Fulop http://orcid.org/0000-0001-5306-6140

Helen Barratt http://orcid.org/0000-0002-1387-137X

For peer review only

REFERENCE LIST

1. National Institute of Clinical Excellence (NICE) Hip, knee and shoulder joint replacement guideline scope, 2018. Available from: https://www.nice.org.uk/guidance/gid-ng10084/documents/draft-scope Accessed Nov 18, 2021.
2. Briggs T. Getting it Right First Time: improving the quality of orthopaedic care within the National Health Service in England, 2012. London, United Kingdom: British Orthopaedic Association.
3. OECD. Hip and knee replacement. Health at a glance, 2019. OECD Indicators. Available from: https://www.oecd-ilibrary.org/sites/2fc83b9a-en/index.html?itemId=/content/component/2fc83b9a-en Accessed Nov 18, 2021.
4. Marsh M, Newman S. Trends and developments in hip and knee arthroplasty technology. *J Rehabil Assist Technol Eng*. 2021;8:2055668320952043. Published 2021 Feb 8. doi:10.1177/2055668320952043
5. Abdelaal MS, Restrepo C, Sharkey PF. Global Perspectives on Arthroplasty of Hip and Knee Joints. *Orthop Clin North Am.* 2020 Apr;51(2):169-176. doi: 10.1016/j.ocl.2019.11.003.
6. Schrijvers G, van Hoorn A, Huiskes N. The care pathway: concepts and theories: an introduction. *Int J Integr Care.* 2012;12(Spec Ed Integrated Care Pathways):e192. Published 2012 Sep 18. doi:10.5334/ijic.812
7. Barbieri A, Vanhaecht K, Van HP et al. Effects of clinical pathways in the joint replacement: A meta-analysis. *BMC Med.* 2009;7:1–11. doi: 10.1186/1741-7015-7-32.
8. Centre for Policy on Ageing. Rapid review: The effectiveness of care pathways in health and social care, 2014. London, United Kingdom: CPA
9. Vanhaecht K, Panella M, van Zelm R et al. An overview on the history and concept of care pathways as complex interventions. *Int. J. Care Pathw.* 2010;14:117–123. doi: 10.1258/jicp.2010.010019.
10. Vanhaecht K. Ph.D. Dissertation. The Katholieke Universiteit Leuven (KU Leuven); Leuven, Belgium: 2007. The Impact of Clinical Pathways on the Organisation of Care Processes.
11. Teisberg E, Wallace S, O'Hara S. Defining and implementing value-based health care: A strategic framework. *Acad. Med*. 2020;95:682–685. doi: 10.1097/ACM.0000000000003122.
12. Berwick DM, Nolan TW, Whittington J. The triple aim: Care, health, and cost. *Health Aff.* 2008;27:759–769. doi: 10.1377/hlthaff.27.3.759.
13. Seys D, Bruyneel L, Deneckere S et al. Better organized care via care pathways: A multicenter study. PLoS ONE. 2017;12:1–11. doi: 10.1371/journal.pone.0180398.
14. Tremblay S-GA, Devitt KS, Kagedan DJ et al. The impact of a clinical pathway on patient postoperative recovery following pancreaticoduodenectomy. HPB (Oxford). 2017;19:799–807. doi: 10.1016/j.hpb.2017.04.015
15. Barratt, H, Turner, S, Hutchings, A, et al. Mixed methods evaluation of the Getting it Right First Time programme - improvements to NHS orthopaedic care in England: study protocol. *BMC Health Serv Res.* 2017 Jan 23;17(1):71. doi: 10.1186/s12913-017-2012-y. PMID: 28115018; PMCID: PMC5260012.
16. Aspinal F, Ledger J, Jasim S, et al. Implementation of the national Getting it Right First Time orthopaedic programme in England: a qualitative case study analysis BMJ Open 2023;13:e066303. doi: 10.1136/bmjopen-2022-066303
17. Getting It Right First Time. Effective Hip and Knee Replacement Pathway, 2020. Available from: https://gettingitrightfirsttime.co.uk/wp-content/uploads/2020/08/GIRFT-Hip-and-Knee-replacement-pathway-May-2020-003.pdf Accessed April 29, 2021.

For peer review only - http://bmjopen.bmj.com/site/about/guidelines.xhtml

18. Van Citters AD, Fahlman C, Goldmann DA, et al. Developing a pathway for high-value, patient-centered total joint arthroplasty. *Clin Orthop Relat Res.* 2014;472(5):1619-1635. doi:10.1007/s11999-013-3398-4
19. Marshall M, Gregory S, & Bullard E, Quality Patient Referrals. *Royal College of General Practitioners* 2018 Available from: https://www.rcgp.org.uk/getmedia/a83fc11f-dd08-4cdd-aefd-b647b03c5611/RCGP-referral-management-feb-2018.pdf
20. Goldsmith LJ, Suryaprakash N, Randall E et al. The importance of informational, clinical, and personal support in patient experience with total knee replacement: a qualitative investigation. *BMC Musculoskelet Disord.* 2017 Dec 24; 18 (1):127. doi: 10.1186/s12891-017-1474-8.
21. Conner-Spady BL, Marshall DA, Hawker GA, et al. You'll know when you're ready: a qualitative study exploring how patients decide when the time is right for joint replacement surgery. *BMC Health Serv Res*. 2014; 14:454. doi: 10.1186/1472-6963-14-454.
22. Sjöling M, Norbergh K-G, Malker H et al. What information do patients waiting for and undergoing arthroplastic surgery want? Their side of the story. *J Orthop Nurs*. 2006;10:5–14
23. Webster F, Perruccio AV, Jenkinson R et al. Where is the patient in models of patient-centred care: a grounded theory study of total joint replacement patients. *BMC Health Serv Res.* 2013 Dec 23;13:531. doi: 10.1186/1472-6963-13-531.
24. Carr T, Teucher UC, Casson AG. Time while waiting: patients' experiences of scheduled surgery. Qual Health Res. 2014 Dec;24(12):1673-85. doi: 10.1177/1049732314549022. Epub 2014 Sep 5.
25. Doyle C, Lennox L, Bell DA. Systematic review of evidence on the links between patient experience and clinical safety and effectiveness. *BMJ Open* 2013;3:e001570. doi: 10.1136/bmjopen-2012-001570
26. Bate P, Robert G. Experience-based design: from redesigning the system around the patient to co-designing services with the patient. *BMJ Quality & Safety* 2006;15:307-310.
27. Barratt H, Hutchings A, Pizzo E et al. Mixed methods evaluation of the Getting it Right First Time programme in elective orthopaedic surgery in England: an analysis from the National Joint Registry and Hospital Episode Statistics. *BMJ Open* 2022;0:e058316. doi:10.1136/bmjopen-2021-058316.
28. Kitzinger J. Qualitative research: Introducing focus groups. BMJ 1995;311:299
29. Focus Group: A Practical Guide for Applied Research. 5th Edition. Richard A. Krueger and Mary Anne Casey. 2015. Sage Publishing ISBN: 978-1-4833-6524-4.
30. Jeffery AE, Wylde V, Blom AW et al. 'It's there and I'm stuck with it': Patients' experiences of chronic pain following total knee replacement surgery. *Arthritis Care Res.* 2011;63:286–92
31. Ledger J, Mehta R, Jasim S et al. O12 Democratising the research process: reflections on the co-production of focus groups. *BMJ Open*, 2019, *9*(Suppl 1), A5–A5. https://doi.org/10.1136/bmjopen-2019-qhrn.12
32. NHS England. Transforming musculoskeletal and orthopaedic elective care services: rethinking referrals (NHSE), 2017. Available from: https://www.england.nhs.uk/wp-content/uploads/2017/11/msk-orthopaedic-elective-care-handbook-v2.pdf Accessed 1 May 2020
33. Brod M, Tesler LE, Christensen TL. Qualitative research and content validity: developing best practices based on science and experience. *Qual Life Res* 2009; 18: 1263–78.
34. Gale NK, Heath G, Cameron E et al. Using the framework method for the analysis of qualitative data in multi-disciplinary health research. *BMC Med Res Methodol* 2013, 13, 117. https://doi.org/10.1186/1471-2288-13-117

For peer review only - http://bmjopen.bmj.com/site/about/guidelines.xhtml

35. British Orthopaedic Association, 2020. NICE Trauma and Orthopaedic Guidelines. Available from: https://www.boa.ac.uk/standards-guidance/nice-trauma-and-orthopaedic-guidelines.html Accessed 15th March 2023
36. Mota REM, Tarricone R, Ciani, O et al. Determinants of demand for total hip and knee arthroplasty: a systematic literature review. BMC Health Serv Res,2012. 12, 225. https://doi.org/10.1186/1472-6963-12-225
37. Sandelowski M. Sample size in qualitative research. Research in Nursing & Health. 1995;18(2):179-183)
38. Punnoose A, Claydon-Mueller LS, Weiss O, Zhang J, Rushton A, Khanduja V. Prehabilitation for Patients Undergoing Orthopedic Surgery: A Systematic Review and Meta-analysis. JAMA Netw Open. 2023;6(4):e238050. doi:10.1001/jamanetworkopen.2023.8050
39. Grocott MP. Pathway redesign: putting patients ahead of professionals. Clin Med (Lond). 2019;19(6):468-472. doi:10.7861/clinmed.2019-0292
40. Carr A, Smith J A, Camaradou J et al. Growing backlog of planned surgery due to covid-19. BMJ 2021;372:n339. doi:10.1136/bmj.n339
41. McCall M, Peebles M, Sandiford NA: Cancellation of elective orthopaedic procedures is not a benign practice and is often preventable. N Z Med J 2021;134:70-78.
42. Caesar U, Karlsson L, Hamrin Senorski E, Karlsson J, Hansson-Olofsson E: Delayed and cancelled orthopaedic surgery; are there solutions to reduce the complex set of problems? A systematic literature review. Int J Clin Pract 2021;75:e14092.

For peer review only

For peer review only - http://bmjopen.bmj.com/site/about/guidelines.xhtml

Figure 1. Eligibility criteria

Figure 2. Patient pathway diagram

For peer review only

For peer review only - http://bmjopen.bmj.com/site/about/guidelines.xhtml

Figure 1: Eligibility criteria

All groups:

- Understand and speak English well enough to be able to give informed consent and participate in a group discussion
- Cognitively able to give informed consent and participate in a group discussion
- Not on a waiting list for total joint arthroplasty (TJA) (in case discussions affect their willingness to undertake TJA surgery)
- Aged over 18 years

Patient groups:

- Undergone elective TKA or THA between March 2016 and March 2018 (to minimise recall bias)
- Received **only** unplanned THA or TKA (because this is a different pathway)

Public group:

- Aged 60 years or older
- have **not** had TJA, but are willing to discuss NHS services to treat joint problems from a public perspective

For peer review only

The Patient Journey: Replacement Surgery

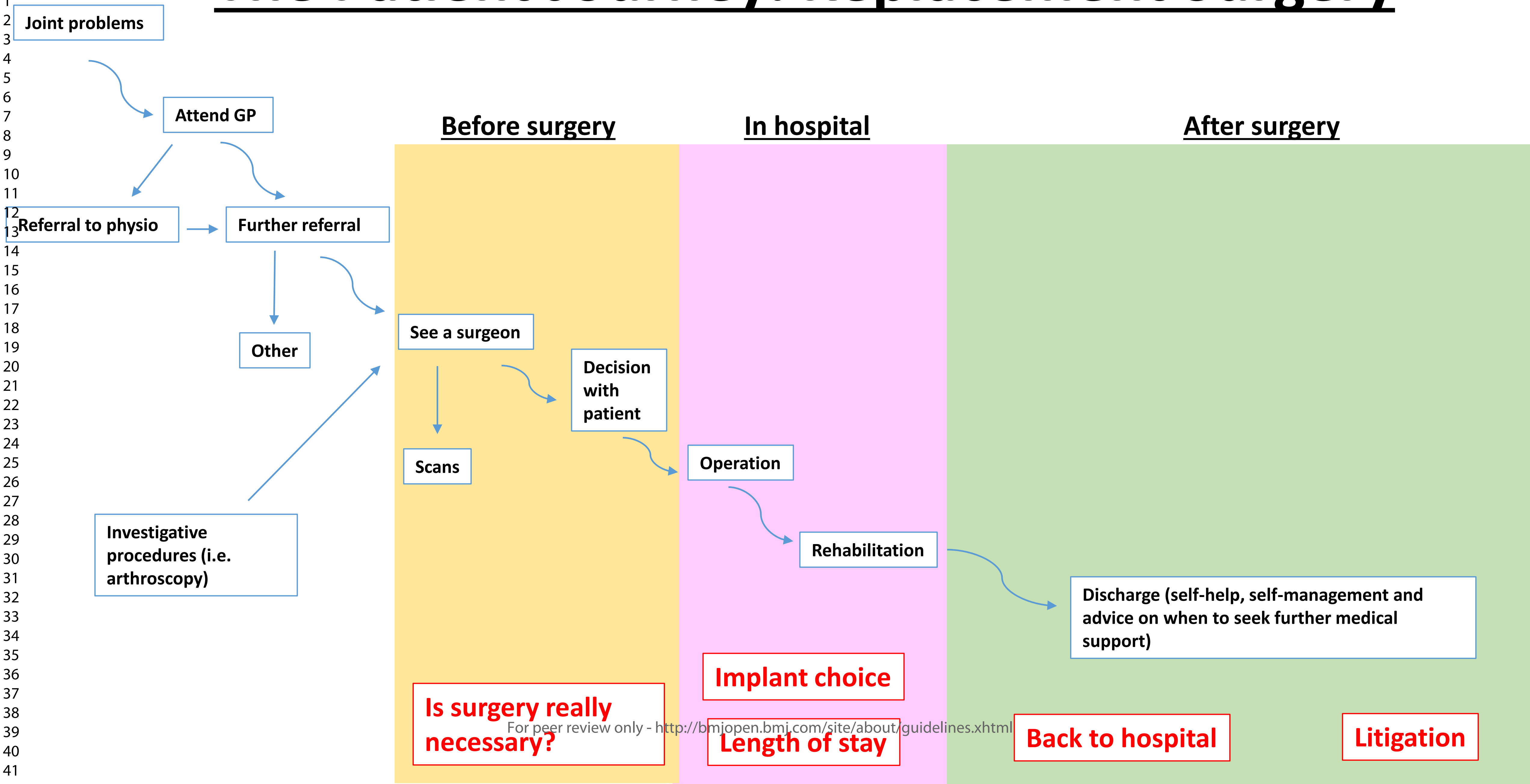

For peer review only - http://bmjopen.bmj.com/site/about/guidelines.xhtml

SUPPLEMENTAL MATERIAL

Online Supplementary File 1: SUMMARY TOPIC GUIDE 1 – GROUPS 1 & 2 (PATIENTS)

SECTION 1: EXPLORING PRACTICE: BEFORE, DURING AND AFTER SURGERY

Looking at this picture of the patient journey, we want to find out what was important to you at different stages:

1. **Before surgery** (orange box):

   At the point at which you were deciding whether to have a hip / knee replacement operation, what was important for you to know about?

2. **In hospital** (pink box):

   Based on your experience of having planned joint surgery in the NHS:

3. **After surgery** (green box):

   Once at home and following surgery, did you need to contact the NHS hospital where you were treated for any reason?

SECTION 2: EXPLORING GIRFT PROGRAMME PRIORITIES

4. In the presentation, we talked about how the GIRFT programme is trying to help hospitals across England provide similar 'results' for all patients who undergo planned orthopaedic surgery (e.g. to improve their life and reduce pain).

5. Are there any GIRFT programme priorities that were covered in the presentation today that we have not discussed and are also important to you?

6. Is there anything else important to you about joint surgery in the NHS, different from the GIRFT programme priorities and that we haven't talked about today?

SECTION 3: CLOSING COMMENTS

7. Finally, if you could give some feedback to the people leading this programme – what might you say?

For peer review only - http://bmjopen.bmj.com/site/about/guidelines.xhtml

Online Supplementary File 2: SUMMARY TOPIC GUIDE 2 – GROUP 3 (PUBLIC INDIVIDUALS)

SECTION 1: EXPLORING PRACTICE: BEFORE, DURING AND AFTER SURGERY

Looking at this picture of the patient journey, we want to find out what is important to you at different stages:

1. **Before surgery** (orange box):

   Scenario: Your GP has recommended that you see an orthopaedic surgeon, as you might need joint replacement surgery (e.g. a hip or knee replacement)

2. **After surgery** (green box):

   Scenario: Imagine you are at home after your hip/ knee replacement operation, and you become concerned about your recovery:

SECTION 2: EXPLORING GIRFT PROGRAMME PRIORITIES

3. In the presentation, we talked about how the GIRFT programme is trying to help hospitals across England provide similar 'results' for all patients who undergo planned orthopaedic surgery (e.g. to improve their life and reduce pain).

4. Are there any GIRFT programme priorities that were covered in the presentation today that we have not discussed and are also important to you?

5. Is there anything else important to you about joint surgery in the NHS, different from the GIRFT programme priorities and that we haven't talked about today?

SECTION 3: CLOSING COMMENTS

6. Finally, if you could give some feedback to the people leading this programme – what might you say?

For peer review only

Online Supplementary File 3: DETAILED STATEMENT FOR REFLEXIVITY

Reflexivity relates to acknowledgment of sensitivity to the ways in which the researchers and the research process may have shaped the data collected, including the role of prior assumptions and experience.[1]

Wider context of the research

Each focus group began with a short presentation explaining the context of the wider 'Getting It Right First Time' (GIRFT) [2] programme in elective orthopaedic surgery in England, and the programme priorities, to ensure all participants had a minimum level of knowledge. During analysis and interpretation, this pre-information was considered, and the possibility of how this knowledge might have shaped what was said.

Professional background of the research team

Members of the research team involved in fieldwork considered the ways in which their interactions with participants might be influenced by their own professional background, experiences, and prior assumptions. Co-facilitators (SJ and JL) were both academic research fellows from non-clinical backgrounds, the patient advisor (RM) who also co-facilitated focus groups had a non-clinical background but had previous experience of non-orthopaedic surgery. The observers (FA and HB) were both senior academic researchers who had clinical backgrounds. During our analysis and interpretation stages we considered whether knowing about our professional backgrounds could have impacted on participants' willingness to openly talk and share their experiences.

Potential for harm or distress

Members of the research team involved in fieldwork were sensitive to the possibility that focusing on the research topic (i.e. recalling previous surgical / in-hospital experiences) could potentially provoke anxiety in the focus group participants concerning the disclosure of adverse events. At the start of each focus group, co-facilitators (RM, SJ, JL) ensured that focus group participants could step out at any time or reveal or disclose as much of their previous experiences and perspectives as they felt comfortable with. At the end of each focus group, the research team stayed behind in the room to ensure that participants had the opportunity to seek support if they were feeling distressed by their participation. No focus group participants expressed such concerns or appeared to be distressed or uneasy.

Collaboration in knowledge production

Collaborative research is valued for its ability to bring together multiple researchers with distinctive and specialist perspectives to tackle large or complex research problems. There was a strong commitment within the research team from the outset to work collaboratively in the collection,

For peer review only

For peer review only - http://bmjopen.bmj.com/site/about/guidelines.xhtml

analysis, interpretation and reporting of the qualitative data, though individual involvement with the various stages of the research process varied as necessary. Team members closely involved in fieldwork (SJ, JL, RM, FA, HB) met frequently on separate occasions to discuss the progress of fieldwork and reflect on data collection. Meetings intensified during the initial stages of data collection and early analysis, when the topic guides and focus group materials were being produced and piloted, and themes and codes were beginning to be identified. At these crucial stages, input was sought from a senior member of the research team with extensive experience of qualitative research (NJF). In the final stages of analysis, input was sought from another senior member of the research team to sense check the coherence, truthfulness, and accuracy of the findings (RR). This resulted in an analytic strategy that was informed by insights from team members with a broad understanding of the research field and methodological issues, and those with field-based contextual and experiential understanding.

For peer review only

For peer review only - http://bmjopen.bmj.com/site/about/guidelines.xhtml

REFERENCE LIST

1. Jootun D, McGhee G, Marland G R. Reflexivity: promoting rigour in qualitative research. *Nurs stand.* 2009;23(23): 42-47.
2. Briggs T. Getting it Right First Time: improving the quality of orthopaedic care within the National Health Service in England, 2012. London, United Kingdom: British Orthopaedic Association.

For peer review only

For peer review only - http://bmjopen.bmj.com/site/about/guidelines.xhtml

Standards for Reporting Qualitative Research (SRQR)*

http://www.equator-network.org/reporting-guidelines/srqr/

Title and abstract

	Page/line no(s).
**Title** - Concise description of the nature and topic of the study Identifying the study as qualitative or indicating the approach (e.g., ethnography, grounded theory) or data collection methods (e.g., interview, focus group) is recommended	1
**Abstract** - Summary of key elements of the study using the abstract format of the intended publication; typically includes background, purpose, methods, results, and conclusions	2

Introduction

**Problem formulation** - Description and significance of the problem/phenomenon studied; review of relevant theory and empirical work; problem statement	4
**Purpose or research question** - Purpose of the study and specific objectives or questions	4

Methods

**Qualitative approach and research paradigm** - Qualitative approach (e.g., ethnography, grounded theory, case study, phenomenology, narrative research) and guiding theory if appropriate; identifying the research paradigm (e.g., postpositivist, constructivist/ interpretivist) is also recommended; rationale**	5
**Researcher characteristics and reflexivity** - Researchers' characteristics that may influence the research, including personal attributes, qualifications/experience, relationship with participants, assumptions, and/or presuppositions; potential or actual interaction between researchers' characteristics and the research questions, approach, methods, results, and/or transferability	6 & Supplement
**Context** - Setting/site and salient contextual factors; rationale**	6
**Sampling strategy** - How and why research participants, documents, or events were selected; criteria for deciding when no further sampling was necessary (e.g., sampling saturation); rationale**	5
**Ethical issues pertaining to human subjects** - Documentation of approval by an appropriate ethics review board and participant consent, or explanation for lack thereof; other confidentiality and data security issues	6
**Data collection methods** - Types of data collected; details of data collection procedures including (as appropriate) start and stop dates of data collection and analysis, iterative process, triangulation of sources/methods, and modification of procedures in response to evolving study findings; rationale**	6

For peer review only - http://bmjopen.bmj.com/site/about/guidelines.xhtml

**Data collection instruments and technologies** - Description of instruments (e.g., interview guides, questionnaires) and devices (e.g., audio recorders) used for data collection; if/how the instrument(s) changed over the course of the study	6
**Units of study** - Number and relevant characteristics of participants, documents, or events included in the study; level of participation (could be reported in results)	5
**Data processing** - Methods for processing data prior to and during analysis, including transcription, data entry, data management and security, verification of data integrity, data coding, and anonymization/de-identification of excerpts	6
**Data analysis** - Process by which inferences, themes, etc., were identified and developed, including the researchers involved in data analysis; usually references a specific paradigm or approach; rationale**	6
**Techniques to enhance trustworthiness** - Techniques to enhance trustworthiness and credibility of data analysis (e.g., member checking, audit trail, triangulation); rationale**	6

Results/findings

**Synthesis and interpretation** - Main findings (e.g., interpretations, inferences, and themes); might include development of a theory or model, or integration with prior research or theory	7-12
**Links to empirical data** - Evidence (e.g., quotes, field notes, text excerpts, photographs) to substantiate analytic findings	7-12

Discussion

**Integration with prior work, implications, transferability, and contribution(s) to the field** - Short summary of main findings; explanation of how findings and conclusions connect to, support, elaborate on, or challenge conclusions of earlier scholarship; discussion of scope of application/generalizability; identification of unique contribution(s) to scholarship in a discipline or field	13
**Limitations** - Trustworthiness and limitations of findings	3 & 13

Other

**Conflicts of interest** - Potential sources of influence or perceived influence on study conduct and conclusions; how these were managed	14
**Funding** - Sources of funding and other support; role of funders in data collection, interpretation, and reporting	14

*The authors created the SRQR by searching the literature to identify guidelines, reporting standards, and critical appraisal criteria for qualitative research; reviewing the reference lists of retrieved sources; and contacting experts to gain feedback. The SRQR aims to improve the transparency of all aspects of qualitative research by providing clear standards for reporting qualitative research.

For peer review only

For peer review only - http://bmjopen.bmj.com/site/about/guidelines.xhtml

**The rationale should briefly discuss the justification for choosing that theory, approach, method, or technique rather than other options available, the assumptions and limitations implicit in those choices, and how those choices influence study conclusions and transferability. As appropriate, the rationale for several items might be discussed together.

Reference:

O'Brien BC, Harris IB, Beckman TJ, Reed DA, Cook DA. **Standards for reporting qualitative research: a synthesis of recommendations.** *Academic Medicine*, Vol. 89, No. 9 / Sept 2014 DOI: 10.1097/ACM.0000000000000388

For peer review only

‘Maze’ not pathway: focus group exploration of patients’ and public experiences of the UK NHS elective total joint arthroplasty pathway.

Journal:	*BMJ Open*
Manuscript ID	bmjopen-2022-066762.R2
Article Type:	Original research
Date Submitted by the Author:	20-Jul-2023
Complete List of Authors:	Jasim, Sarah; The London School of Economics and Political Science, Care Policy and Evaluation Centre<br>Aspinal, Fiona; University College London, Department of Applied Health Research<br>Mehta, Raj; University College London, Department of Applied Health Research<br>Ledger, Jean; University College London<br>Raine, Rosalind; University College London, Institute of Epidemiology & Health<br>Fulop, Naomi; University College London, Applied Health Research<br>Barratt, Helen; UCL, Department of Applied Health Research
**Primary Subject Heading**:	Health services research
Secondary Subject Heading:	Qualitative research, Surgery
Keywords:	Orthopaedic & trauma surgery < SURGERY, QUALITATIVE RESEARCH, Hip < ORTHOPAEDIC & TRAUMA SURGERY, Knee < ORTHOPAEDIC & TRAUMA SURGERY, Quality in health care < HEALTH SERVICES ADMINISTRATION & MANAGEMENT

I, the Submitting Author has the right to grant and does grant on behalf of all authors of the Work (as defined in the below author licence), an exclusive licence and/or a non-exclusive licence for contributions from authors who are: i) UK Crown employees; ii) where BMJ has agreed a CC-BY licence shall apply, and/or iii) in accordance with the terms applicable for US Federal Government officers or employees acting as part of their official duties; on a worldwide, perpetual, irrevocable, royalty-free basis to BMJ Publishing Group Ltd ("BMJ") its licensees and where the relevant Journal is co-owned by BMJ to the co-owners of the Journal, to publish the Work in this journal and any other BMJ products and to exploit all rights, as set out in our licence.

The Submitting Author accepts and understands that any supply made under these terms is made by BMJ to the Submitting Author unless you are acting as an employee on behalf of your employer or a postgraduate student of an affiliated institution which is paying any applicable article publishing charge ("APC") for Open Access articles. Where the Submitting Author wishes to make the Work available on an Open Access basis (and intends to pay the relevant APC), the terms of reuse of such Open Access shall be governed by a Creative Commons licence – details of these licences and which Creative Commons licence will apply to this Work are set out in our licence referred to above.

Other than as permitted in any relevant BMJ Author's Self Archiving Policies, I confirm this Work has not been accepted for publication elsewhere, is not being considered for publication elsewhere and does not duplicate material already published. I confirm all authors consent to publication of this Work and authorise the granting of this licence.

For peer review only

‘Maze’ not pathway: focus group exploration of patients’ and public experiences of the UK NHS elective total joint arthroplasty pathway.

*Sarah Jasim, Care Policy and Evaluation Centre, London School of Economics and Political Science, Houghton Street, Holborn, London, WC2A 2AE, UK s.jasim@lse.ac.uk

Sarah Jasim, Care Policy and Evaluation Centre, London School of Economics and Political Science, UK

Fiona Aspinal, Department of Applied Health Research, University College London, UK

Raj Mehta, Patient Advisor, NIHR ARC North Thames Research Advisory Panel, Department of Applied Health Research, University College London, UK

Jean Ledger, Department of Applied Health Research, University College London, UK

Rosalind Raine, Department of Applied Health Research, University College London, UK

Naomi J. Fulop, Department of Applied Health Research, University College London, UK

Helen Barratt, Department of Applied Health Research, University College London, UK

*corresponding author

Word count: 3261 words

For peer review only

For peer review only - http://bmjopen.bmj.com/site/about/guidelines.xhtml

ABSTRACT

Objective: To explore patient and public perceptions of planned improvements to the NHS TJA pathway.

Design: Three qualitative focus groups undertaken March – May 2018, as part of a mixed-methods evaluation of GIRFT. Transcripts were subject to framework analysis to identify thematic content between October 2018 – October 2021.

Setting: Elective TJA surgery in the English NHS.

Participants: Two focus groups including patients who had undergone TJA in the previous two years (Group 1: n=5; Group 2: n=4) and the other individuals who had not but were aged 60+ (Group 3: n= 5). Participants were recruited via community groups and patient panels.

Results: Fourteen individuals took part in the focus groups; all were aged over 60; seven (50%) were female; and nine (64%) had undergone TJA surgery. Participants' perspectives were categorised into themes and mapped onto stages of the TJA pathway. Although perioperative care is often the focus of improvement efforts, participants argued that the patient journey begins before individuals present to primary care. Participants had concerns about other aspects of the pathway, such as obtaining a surgical referral, with pre-referral interventions aimed at potentially avoiding the need for surgery (i.e., physiotherapy) being perceived as a mechanism to restrict access to secondary care. Patient experience was also conceptualised as a 'maze', rather than the logical, sequential process set out in clinical guidelines; exacerbated by a lack of information, knowledge, and power imbalances.

Conclusion: The linear conceptualisation of the TJA pathway is at odds with patient experience. Improvement programmes focussed on perioperative care fail to consider patient concerns and priorities. Patients should be directly involved in improvement programmes, to ensure that patient experience is optimised, as well as informing related processes and important outcomes of care.

For peer review only

STRENGTHS AND LIMITATIONS OF THIS STUDY

- This is the first study to look at the entire TJA pathway from the perspective of patients and the public and explore how their priorities differ from those of clinicians and managers seeking to improve care.
- Co-producing research with a patient advisor ensured the patient perspective was central to our work from planning through to analysis and dissemination.
- We sought to recruit via a wide range of different channels, including professional societies, patient groups, and local community groups (including multi-faith organisations).
- Nevertheless, we were not able to recruit a diverse sample with respect to ethnicity, and one participant withdrew because of fears of cultural stigma.
- As participants volunteered to participate, there may be a degree of self-selection bias, with participants who were more confident in articulating their views and experiences or indeed, those with particular types of experiences, potentially more likely to participate.

For peer review only

For peer review only - http://bmjopen.bmj.com/site/about/guidelines.xhtml

BACKGROUND

Elective hip and knee replacement are the two most common total joint arthroplasty (TJA) procedures, and two of the most frequently performed and effective surgical procedures in the UK [1,2] and globally.[3] With an increasingly ageing population, and trend for TJA earlier in life,[4] the predicted growth in global demand is likely to have a significant impact on health systems,[5] such as the National Health Service (NHS) in England.

A care pathway [6] is a multidisciplinary tool for planning the care of specific, well-defined groups of patients who have a predictable clinical course, such as TJA. Drawing on research evidence and best practice, it sets out how different components of care should be optimised and sequenced, to enhance quality [7] and improve patient safety. This approach has been shown to reduce unnecessary variation [6,8-11] and may improve patient outcomes and optimise resource use.[6,12-14] Standardisation of care is important for national improvement programmes such as the English 'Getting It Right First Time' programme (GIRFT), which aims to tackle unwarranted variation in orthopaedic surgery across the NHS.[15,16] GIRFT sought to develop a 'best of the best' clinical pathway [17] to improve patient experience and expedite recovery following TJA. Although clinical perspectives are central to pathway development,[8] the views of current and future patients who must navigate them, are equally important to optimise experience, outcomes, and efficiency.[18]

In the UK, general practitioners (GPs) act as gatekeepers to secondary care, including the TJA pathway. [19] Patients experiencing hip or knee pain typically first present to a GP. In some English regions, local commissioners (leaders responsible for purchasing health care on behalf of local communities) are increasingly setting criteria that patients must meet before they can be referred to an orthopaedic surgeon. Current guidance recommends conservative management initially, for at least three months, with the aim of potentially avoiding the need for the patient to undergo surgery. Interventions include medication, physiotherapy, and/or support with lifestyle and weight loss. If this is not successful, the GP should refer the patient to an orthopaedic surgeon to discuss potential surgery.

Existing research about patient perspectives of the TJA pathway has tended to focus on patient satisfaction and experience [20] at specific stages of the pathway, such as deciding to undergo surgery,[21] pre-operative education,[22] waiting for surgery,[23-24] and management of post-operative pain.[12] Given shared decision-making is considered integral to care pathways as well as wider efforts to improve care, there is currently no evidence on how patients and public view planned improvements to the TJA pathway, and whether their priorities align with those of clinicians and managers.[25,26] This study sought to address that gap by exploring patient and public perceptions of planned improvements to the TJA pathway, including the factors that influenced this. The research was part of a wider mixed methods evaluation of the GIRFT programme.[15,16,27]

For peer review only - http://bmjopen.bmj.com/site/about/guidelines.xhtml

METHODS

Study Design and Recruitment

Focus groups enabled us to collect views from multiple participants simultaneously and explore meanings behind those views, which were generated, discussed, and refined through group interactions in a way that could not happen with other methods.[28]

To explore the outcome of interest, which was patient and public perceptions of planned improvements to the NHS TJA pathway, we sought to recruit between four to eight participants to each of three focus groups. This was based on evidence regarding the optimal number of groups and participants for focus group research.[29] Groups 1 and 2 included patients who had undergone an elective hip or knee replacement in the previous two years (March 2016–March 2018). Group 3 included individuals who had not undergone TJA but were in the age bracket most likely to receive this type of intervention (age 60+) (Figure 1).[30] This enabled us to compare experiences of those who had undergone TJA with the expectations of those who had not, and how this influenced participants' views. In this paper we report the findings from all three groups together, referring to individuals as participants, except where there were clear differences between those who had undergone surgery, and those who had not. We recruited participants via community organisations across London (UK) whose membership was likely to be aged 60 and over (e.g., third sector organisations, day centres and activity clubs). We also invited participation from eligible patient advisory group members from relevant organisations (e.g., the British Orthopaedic Association). Researchers (SJ, JL) conducted telephone and e-mail screening and collected preliminary demographic information to maximise sample diversity (Table 1).

Table 1: Participant characteristics

	**Group 1**<br>(Patients – n=5)	**Group 2**<br>(Patients – n=4)	**Group 3**<br>(Public – n=5)
**Sex**	Male (n=2), Female (n=3)	Male (n=2), Female (n=2)	Male (n=3), Female (n=2)
**Age range**	60-69 (n=2)<br>70-79 (n=3)	60-69 (n=1)<br>70-79 (n=1)<br>80-89 (n=2)	60-69 (n=3)<br>70-79 (n=2)
**Ethnicity**	White British (n=4)<br>White European (n=1)	White British (n=3)<br>White South African (n=1)	White British (n=4)<br>White North American (n=1)

For peer review only - http://bmjopen.bmj.com/site/about/guidelines.xhtml

Data Collection

We conducted three focus groups between March to May 2018 in an academic (non-healthcare) setting. Observation data were collected by senior researchers (FA or HB) to capture any contextual factors that might have impacted data quality, such as the topics being discussed or depth of discussion. All participants gave informed consent. Each focus group began with a brief presentation by the research team, providing a high-level overview of efforts to improve the TJA pathway for patients, including GIRFT.[15]

Focus group discussions were informed by semi-structured topic guides (online supplementary file 1-2), which included participants' priorities before, during and after surgery, as well as their views of the planned improvements to the TJA pathway. Each group lasted approximately two hours and was audio-recorded using an encrypted device for professional transcription in full.

Patient and Public Involvement

Three focus groups were co-facilitated by a researcher (SJ or JL) and the patient advisor (RM).[31] Following advice from our patient advisor (RM),[31] we (SJ) also provided participants with a simplified illustration of the UK TJA care pathway (Figure 2), based on clinical guidance at the time.[32] The pathway diagram and topic guides were piloted with a research advisory panel of patient advisers and refined initially, and then iteratively as the research progressed (i.e., diagram title and emphasis of questions), to take account of emerging findings, in line with accepted qualitative methods.[33] Preliminary results of the study were verbally presented to participating patient advisors.

Data Analysis

The core research team (HB, FA, RM, SJ, JL) held de-brief meetings after each focus group to ensure triangulation between focus group findings and researcher observations. Observation notes and field notes were not subject to formal analysis but were used in debrief and analysis meetings to ensure transcript data were contextualised. Transcript data were analysed using a Framework approach.[34] We used the care pathway diagram (Figure 2) and structure of the topic guides (online supplementary file 1-2) to develop the initial coding framework, and incorporated concepts and meanings identified in the data.[13]

The first stage of analysis involved familiarisation, where researchers (SJ, JL, FA, HB) read and collaboratively reviewed transcripts to understand meanings, concepts, and ideas, in a reflexive manner (see online supplementary file 3). The wider research team (SJ, JL, RM, FA, HB, NJF) then identified and confirmed parameters of the main themes to form the basis of a thematic framework in a series of separate meetings. Data were indexed based on the thematic framework using MAXQDA software (SJ). Indexed data were organised into thematic charts for each category, in Microsoft Excel, using direct quotations where possible. These were reviewed and modified when necessary to clarify meaning (FA). Themes were further mapped and interpreted to understand areas of significance to patients and the public (SJ, FA, HB, JL, RM, NJF, RR).

Ethics

Ethical approval was granted by NHS North West – Liverpool East Research Ethics Committee (16/NW/0654).[15]

For peer review only - http://bmjopen.bmj.com/site/about/guidelines.xhtml

RESULTS

Participants

Seventeen individuals agreed to participate, although three withdrew on the day. Two because of heavy snowfall (Group 2 and Group 3) and one due to fears of cultural stigma from openly discussing experiences (Group 2). All 14 participants were aged over 60 and all self-identified as White British or White Other. Seven were female, and 9 participants had undergone TJA surgery in the previous two years. Of the 5 who had not undergone surgery (i.e., Group 3), 3 had friends or family who had previously undergone TJA.

Our original goal was to explore participants' views about planned improvements to the TJA pathway (such as GIRFT),[2,15] which were focused particularly on the perioperative 'in-hospital' (Figure 2) phase of the care pathway. However, it quickly became apparent that other phases of the pathway were of equal, if not more, importance to participants. Participants understood the pathway diagram, but argued that whilst the streamlined, linear presentation may be how it appears to clinicians and managers, it did not reflect their experiences. Here, we report participants' views about priorities for pathway improvement; the realities of traversing the TJA pathway; and barriers and facilitators they encountered whilst navigating the pathway (participant quotations are provided in Table 2).

For peer review only

For peer review only - http://bmjopen.bmj.com/site/about/guidelines.xhtml

Table 2: Focus group (FG) quotes

Ref.	FG No.	Gender	Quote
Quote1	1	Male	'…… that's [pointing to the first part of the pathway diagram] probably where most problems, as far as I'm concerned, arose. In that trying to see a consultant was very difficult.'
Quote2	1	Male	'…I get a letter in the post saying, it's a confirmation of the date, so that would be followed by another letter that says they've just cancelled that appointment…With no explanation, it just says it's cancelled… "What happens, where are we now, or what happens now?"…they said, "Oh well, it's got to go in before the assessment committee"… Well, this is very, very … upsetting that… I'm in a lot of pain, I want to see a consultant, and there's all these obstacles in the way before you can even see the consultant.'
Quote3	1	Female	'I had months of physiotherapy right through till February the following year. It wasn't helping, I didn't think it was helping a lot... And then in the February I spoke to my physiotherapist, asked him… Am I going to go on and on like this? … So, I'm now thinking, "…they're just… shuffling me along," obviously … would I like to just go ahead with the op…'
Quote4	1	Male	'Yeah, I think it's a postcode lottery, isn't it, where some people live that there's a huge waiting list and I think the GPs…refer them to the physiotherapist first so… keeps them out of the way for a bit…'
Quote5	2	Male	'…the GP is the stopping point, and it causes a lot of the other things going up the line to be worse than they need to be because they resist. Obviously, they're the Clinical Commissioning Group that actually drives them on this and say, 'Don't refer, don't refer, don't refer, we'll even give you some extra bonus points if you don't refer.'
Quote6	2	Male	'…so it was down to you to look after your own, and when it all starts to go wrong, you've got to go to your GP and you've got to persuade him to refer you to a specialist…'
Quote7	1	Female	'…I felt I had to bully them in order to have the surgery…'
Quote8	1	Female	'– I was just taken aback by how much I felt like a widget on a production line.'
Quote9	3	Female	'…if you like, you'd need a referral to Social Services. I think that should be part of the pre-operative planning rather than suddenly realising afterwards. What I was talking about was the OT assessment and the follow-up physiotherapy, I wasn't talking about Social Services, about social care support, which you may well need and that should have really been part of the planning beforehand. Because if you've got to wait 'til afterwards you'll be in hospital for a month before anything happens. I know because I've been a social worker so, you know, I know what it's like.'
Quote10	1	Female	'lack of communication between various people, the doctors, the nurses, so that I felt I was the person who knew more than anyone else about what was going on, but I knew least about what should be going on'
Quote11	3	Female	'I'd like to know about physiotherapy and after care and the system they use…So I'd want to know, if I was going to go in, whether I'd be not only would the surgery be good and efficient but there would be a very good programme coming out in after care and who'd be doing that with me.'
Quote12	3	Female	'I want to come back to the Social Services and what happens when you get out of hospital, especially if you live alone or... you know, how do you manage for that period of time after hospital, that's an important handover and that's not always handled really well.'
Quote13	1	Male	'..like a maze'
Quote14	1	Male	'all the time there's never any communication as to what's happening. So, you're left in the dark, and that itself is upsetting because, you know, you want to get it dealt with, you don't know at what stage it's going to happen, and you're just being pushed from pillar to post, and nobody will give you any information.'
Quote15	3	Female	'There's a big thing about sort of communication through the line so that people don't rely on you as the rather disorientated patient after an operation to actually be the person who's informing so that there are other people sharing information, but you've also got a voice, that your voice is going to be heard or that you've elected someone to speak for you and that person's voice is going to be heard as well.'
Quote16	3	Male	'information tends to be... You're always given something when you go out - and this is all kinds of hospitalisation, not just surgery… but it's never quite enough… but it's generally not good…'

For peer review only - http://bmjopen.bmj.com/site/about/guidelines.xhtml

Quote17	1	Female	'because I can see from this chart [diagram] that I'm on a conveyor belt and that's the feeling that I had. And actually, I felt that I'd been in the dark from the start. And I felt that there is information there if I ask the right question, but I don't know what question to ask. And I have felt that I have to look it up a lot myself, I have to find out things myself'
Quote18	2	Female	"Well, personally I wasn't aware that there was even any choice."
Quote19	3	Female	'It is actually a plus point for hospitals to make their statistics available publicly in some way because people will search for excellence and people will avoid mediocrity, and I certainly don't depend on my GP for that...'
Quote20	1	Female	'So, that question of choice actually goes all the way through. Is it really choice or not and how much power do we really have? How much knowledge do we have?'
Quote21	1	Female	"I was so intimidated by him, and so overwhelmed by him, I thought, 'Right, okay, well, you know, I'm just going to … there's no point in my doing anything else here'."
Quote22	3	Female	'…care-givers are important too and I can't tell you how many times I was at a doctor and the doctor just ignored the questions that I had to ask because I was not the patient...'
Quote23	1	Female	'But it was about two weeks before my operation, so if you are anxious and you feel like you're in the dark, that's a long time to wait before you get those questions, a chance to answer those questions, and you find out new information. It was at the joint school that I found out that you don't have a general anaesthetic, that you have a spinal anaesthetic.'
Quote24	1	Female	'But then physio told me that there was a system whereby you get a choice of hospitals for having your operation in. I didn't know that either. She said, 'Haven't you heard about this before?' And she gave me a list of six hospitals, the physio… she said, 'Look them up, go and read the CQC report on these hospitals and see which one you like, and if you prefer one of the other ones, come back and let me know'. I'm sorry, having come through all this and been so in the dark I'm now being told to decide on what hospital.'
Quote25	1	Female	'I knew the system more, I was much more able to work my way through it, to talk to the right people, to make the decision'
Quote26	3	Female	'…I'd want the occupational therapist to assess me, assess my home, work out what the … support I'm going to get at home to actually help me achieve mobility and how accessible they are…'

Patient priorities: obtaining a referral to see an orthopaedic surgeon

Although the peri-operative phase of the pathway is often the focus of improvement programmes, such as GIRFT, individuals who had undergone TJA argued that obtaining a referral to an orthopaedic surgeon is in fact a greater challenge and concern (see Table 2, Quote1). This was 'upsetting' given their level of pain or disability (Quote2) especially when appointments were postponed. Some participants found that GP-recommended non-surgical interventions resulted in little or no improvement, whilst others had been in too much pain to engage with physiotherapy (Quote3). Consequently, they viewed this approach simply as a mechanism for managing demand (Quote4). Some participants believed that GPs had pressure placed on them to not refer patients to secondary care (Quote5,). Others felt that external pressures to ration NHS services impacted their access, such as referrals being reviewed by an 'assessment committee' (Quote2) or appointments being delayed by repeated, unexplained cancellations.

Because of the obstacles they had faced, participants spoke about the difficulties encountered to get onto the pathway and secure an orthopaedic referral, and in some cases, 'persuade' (Quote6-) or even 'bully' (Quote7-) healthcare professionals, whilst on this conveyor belt' (Quote17) or 'production line' (Quote8).

The realities of the elective TJA pathway

For peer review only - http://bmjopen.bmj.com/site/about/guidelines.xhtml

Participants, especially those who had not undergone surgery (Group 3), emphasised the importance of joined-up care planning throughout the pathway, including clinical teams planning surgery dates to coincide with available post-operative support (Quote9). Disconnected communication and ways of working between health providers at different stages of the pathway, left patients (Groups 1 and 2) having to assume responsibility for their own care, despite feeling powerless with insufficient information to make such informed decisions (Quote10). The lack of joined-up care was important to all groups and seen as being exacerbated by poor communication. For example, they described poor discharge procedures where communication with wider services was lacking, such as pharmacy, community health services, rehabilitative physiotherapy (Quote11) and social care (Quote12).

Navigating the 'maze'

Without joined-up forward-planning, patients had to manage and navigate a range of complex local health systems alone. They were left feeling adrift from the system, and many participants felt they were ill-equipped to tackle the obstacles they encountered because of asymmetries of both information and power. As one neatly summarised, the pathway is 'like a maze' (Quote13).

Lack of information and knowledge

Participants reported that from the point of referral from primary care, they were 'left in the dark' (Quote14). A lack of communication (Quote15) or appropriate, timely information from their healthcare provider hindered their understanding of the process (, , Quote16) making it difficult for them to make informed choices . This was compounded for some by a lack of knowledge, meaning that they felt they did not know what questions they should be asking. This was particularly a concern in the stages prior to surgery but differed according to participants' direct or indirect experiences; location of care; and pre-existing knowledge or professional background (Quote17).

Although clinical guidance [35] implies shared decision-making should be built into the pathway, participants' knowledge about available choices varied. For example, some participants in all groups had not known that they could choose the hospital they went to, or their surgeon, whilst others had sought out information to inform their choice (Quote18, Quote19). Nevertheless, even those who were aware that they could exercise choice, felt that they lacked sufficient knowledge to do so (Quote20).

Power imbalance

Alongside a lack of information and communication, many participants described how they had to disrupt existing power dynamics to navigate the TJA pathway. Asymmetries of power in relationships with key health professionals could significantly impact their experience of care (Quote8) and their progress along the pathway. For example, negative interactions with a surgeon had halted one participant's journey during their first appointment with a surgeon and hindered their access to pathway at the very start (Quote21). Another participant, who had cared for a TJA patient, described how her attempts to advocate for them were ignored (Quote22).

Overcoming obstacles

Participants described three distinct sources of knowledge which they drew on to differing degrees to overcome the obstacles they faced: formal learning; informal learning; and lived experience.

Formal learning

For peer review only - http://bmjopen.bmj.com/site/about/guidelines.xhtml

Formal learning opportunities offered to patients prior to surgery had helped some. For example, pre-operative education groups (also known as 'Joint Schools') run by hospital staff for patients listed for surgery, had alleviated fears, increased knowledge, and provided an opportunity to ask questions. Nevertheless, for some, the programme was offered very close to the date of the procedure, meaning that they had a long wait to raise their concerns. Consequently, some would have liked this to have been offered much earlier in the pathway (Quote23).

Informal learning

Some participants also sought out or expected to seek out information for themselves that they thought they needed to navigate the process. This included details about medication (carer, Group 3); chasing appointments (female patient, Group 1); and undertaking their own research to inform choices (all groups) and guide decision-making (female patient, Group 1). Others had also sought information from health professionals such as physiotherapists who were familiar with the TJA pathway, which helped them feel 'less in the dark' (Quote24).

Lived experience

Participants who were able to draw on a previous experience of undergoing surgery, used this to help inform choices and decision-making and felt better able to navigate the pathway on subsequent occasions. Those who had previously undergone a TJA knew to request additional information about regaining function, resuming activities, and exercising, not only to improve outcomes but also to assist navigating the crucial post-operative stage of the pathway (Quote26). Some also believed that this knowledge had enabled them to expedite the process on subsequent occasions, including identifying potential short cuts (Quote25,).

For peer review only - http://bmjopen.bmj.com/site/about/guidelines.xhtml

DISCUSSION

Despite ongoing efforts to streamline and standardise care, patient experience differs significantly from the seemingly logical, sequential process set out in clinical guidelines. [35] Importantly for patients, the pathway does not begin and end at the doors of the hospital. For example, it starts at first presentation to primary care with joint pain. A lack of timely information and communication also leaves patients feeling that they have been 'left in the dark' to navigate a 'maze'. This sense is exacerbated by power and information asymmetries between patients and clinicians. Whilst formal education programmes such as 'Joint School' can be helpful, many patients have to seek out additional information for themselves to answer their queries, understand the procedure and navigate the TJA pathway.

Previous research has largely focused on specific phases of the care pathway. This is the first study to look at the entire TJA pathway from the perspective of patients and the public and explore how their priorities differ from those of clinicians and managers seeking to improve care. Co-producing research with a patient advisor ensured the patient perspective was central to our work from planning through to analysis and dissemination. Qualitative data collection methods were used to allow participants to describe their experience and views in their own words, and the focus group approach enabled sharing, comparing and corroboration of ideas. As socio-economic circumstances may impact a patient's decision to undergo TJA,[36] we sought to recruit via a wide range of different channels, including professional societies, patient groups, and local community groups (including multi-faith organisations). Nevertheless, we were not able to recruit a diverse sample with respect to ethnicity. Indeed, one participant withdrew because of fears of cultural stigma. It is acknowledged that cultural differences may exist between other groups of patient and public members. The study methodology and number of study participants may limit the transferability of findings – although this was mitigated by using purposeful sampling there was geographical variability in participants' lived experiences of the UK TJA pathway. [37] The methodology was selected to explore a range of views in depth, and not necessarily representation. As participants volunteered to participate, there may be a degree of self-selection bias, with participants who were more confident in articulating their views and experiences or indeed, those with particular types of experiences, potentially more likely to participate.

Our findings have several implications for clinicians and policymakers. Although perioperative care is often the focus of clinically led efforts (e.g., GIRFT) [2,27] to improve the TJA pathway, participants also emphasised that obtaining a referral to a surgeon is often the greatest challenge, and the post-operative period following surgery is a priority. Despite some evidence to suggest that prehabilitation is associated with improving some of the outcomes for patients undergoing orthopaedic surgery both preoperatively and postoperatively, we have highlighted that non-surgical interventions (e.g., physiotherapy) prior to surgical referral are perceived by patients and the public as a means of managing demand. [38] While this remains the case, as well as potentially damaging patient trust, it is likely to impact patient engagement with evidence-based care. Efforts to provide patients with information about their procedure and likely recovery (e.g., via 'Joint Schools') are welcome, but many want information about the pathway earlier than this so that they are not left feeling ill-equipped to navigate the 'maze' of care themselves, especially if they have not previously undergone surgery. At a policy-level, those working to standardise services using care pathways should also be aware that patient experience is seldom analogous to their linear, stepwise conceptualisations. In line with the existing literature, our findings suggest that experience-based co-design should be a core part of pathway re-design.[39] Challenges with traversing the TJA pathway, alongside the negative impacts on physical and mental wellbeing of delayed appointments that were reported in this study, are likely to be exacerbated by the COVID-19 pandemic which has resulted in

For peer review only - http://bmjopen.bmj.com/site/about/guidelines.xhtml

further delays, cancellations, and increased waiting times for those on the TJA pathway. [40] The subsequent impacts on the health system have increased the length of patients' experience of pain, decreased mobility, worsened quality of life. [41,42]

CONCLUSION

In conclusion, we have highlighted the disparity between the priorities of improvement programmes such as GIRFT and those of patients and the public. Whilst improvement programmes typically seek to improve peri-operative care, patient concerns lie elsewhere, for example obtaining a referral to an orthopaedic surgeon and managing recovery at home. Research that includes views from both patients and health care professionals is needed at these crucial stages of the TJA pathway to explore how patients are being supported. The typically linear conceptualisation of TJA pathways in policy documents is also at odds with how patients experience the process. With rates of TJA set to increase globally, patients and public must be directly involved in improvement programmes to optimise not only processes and outcomes of care, but also patient experience.

For peer review only

For peer review only - http://bmjopen.bmj.com/site/about/guidelines.xhtml

DECLARATIONS

Acknowledgements: The authors would like to acknowledge and thank the focus group participants, and especially Steven Towndrow, Wendy Chandler, Stephanie Hume, Patricia Hallam and the NIHR ARC (formerly CLAHRC) North Thames Research Advisory Panel patient advisers for their efforts in preparation and organisation of the focus groups.

Author Contributions: HB was principal investigator. HB and RR initiated the research. HB, NJF and RR designed the evaluation. SJ, RM and JL were responsible for qualitative data collection; SJ, FA,RM, JL, NJF and HB analysed the qualitative data. SJ, FA, RM, JL, RR, NJF and HB drafted the manuscript and all authors contributed to reviewing and substantive revision. All authors approved the final version. All authors had full access to all the data in the study and accept responsibility to submit for publication. HB will act as guarantor.

Funding: National Institute for Health and Care Research (NIHR) Programme Grants for Applied Research (Applied Research Collaboration (ARC) North Thames)

Disclaimer: This report is independent research funded by the National Institute for Health Research ARC North Thames. The views expressed in this publication are those of the authors and not necessarily those of the National Institute for Health Research or the Department of Health and Social Care.

Competing interests: No competing interests.

Ethics approval and consent to participate: This study involved human participants and was reviewed and approved by NHS North West - Liverpool East Research Ethics Committee (16/NW/0654). Before taking part, all participants gave informed consent to participate in the study and for be analysed and reported anonymously.

Provenance and peer review: Not commissioned; externally peer reviewed.

Data availability statement: No data are available.

Consent for publication: Not applicable

Availability of data and materials: Consent was not given by participants to share their anonymised transcripts.

For peer review only

ORCID iDs:

Sarah Jasim http://orcid.org/0000-0003-3940-6350

Fiona Aspinal http://orcid.org/0000-0003-3170-7570

Raj Mehta http://orcid.org/0000-0002-4003-530X

Jean Ledger http://orcid.org/0000-0003-2523-7971

Rosalind Raine http://orcid.org/0000-0003-0904-749X

Naomi J Fulop http://orcid.org/0000-0001-5306-6140

Helen Barratt http://orcid.org/0000-0002-1387-137X

For peer review only

For peer review only - http://bmjopen.bmj.com/site/about/guidelines.xhtml

REFERENCE LIST

1. National Institute of Clinical Excellence (NICE) Hip, knee and shoulder joint replacement guideline scope, 2018. Available from: https://www.nice.org.uk/guidance/gid-ng10084/documents/draft-scope Accessed Nov 18, 2021.
2. Briggs T. Getting it Right First Time: improving the quality of orthopaedic care within the National Health Service in England, 2012. London, United Kingdom: British Orthopaedic Association.
3. OECD. Hip and knee replacement. Health at a glance, 2019. OECD Indicators. Available from: https://www.oecd-ilibrary.org/sites/2fc83b9a-en/index.html?itemId=/content/component/2fc83b9a-en Accessed Nov 18, 2021.
4. Marsh M, Newman S. Trends and developments in hip and knee arthroplasty technology. *J Rehabil Assist Technol Eng*. 2021;8:2055668320952043. Published 2021 Feb 8. doi:10.1177/2055668320952043
5. Abdelaal MS, Restrepo C, Sharkey PF. Global Perspectives on Arthroplasty of Hip and Knee Joints. *Orthop Clin North Am.* 2020 Apr;51(2):169-176. doi: 10.1016/j.ocl.2019.11.003.
6. Schrijvers G, van Hoorn A, Huiskes N. The care pathway: concepts and theories: an introduction. *Int J Integr Care.* 2012;12(Spec Ed Integrated Care Pathways):e192. Published 2012 Sep 18. doi:10.5334/ijic.812
7. Barbieri A, Vanhaecht K, Van HP et al. Effects of clinical pathways in the joint replacement: A meta-analysis. *BMC Med.* 2009;7:1–11. doi: 10.1186/1741-7015-7-32.
8. Centre for Policy on Ageing. Rapid review: The effectiveness of care pathways in health and social care, 2014. London, United Kingdom: CPA
9. Vanhaecht K, Panella M, van Zelm R et al. An overview on the history and concept of care pathways as complex interventions. *Int. J. Care Pathw.* 2010;14:117–123. doi: 10.1258/jicp.2010.010019.
10. Vanhaecht K. Ph.D. Dissertation. The Katholieke Universiteit Leuven (KU Leuven); Leuven, Belgium: 2007. The Impact of Clinical Pathways on the Organisation of Care Processes.
11. Teisberg E, Wallace S, O'Hara S. Defining and implementing value-based health care: A strategic framework. *Acad. Med*. 2020;95:682–685. doi: 10.1097/ACM.0000000000003122.
12. Berwick DM, Nolan TW, Whittington J. The triple aim: Care, health, and cost. *Health Aff.* 2008;27:759–769. doi: 10.1377/hlthaff.27.3.759.
13. Seys D, Bruyneel L, Deneckere S et al. Better organized care via care pathways: A multicenter study. PLoS ONE. 2017;12:1–11. doi: 10.1371/journal.pone.0180398.
14. Tremblay S-GA, Devitt KS, Kagedan DJ et al. The impact of a clinical pathway on patient postoperative recovery following pancreaticoduodenectomy. HPB (Oxford). 2017;19:799–807. doi: 10.1016/j.hpb.2017.04.015
15. Barratt, H, Turner, S, Hutchings, A, et al. Mixed methods evaluation of the Getting it Right First Time programme - improvements to NHS orthopaedic care in England: study protocol. *BMC Health Serv Res.* 2017 Jan 23;17(1):71. doi: 10.1186/s12913-017-2012-y. PMID: 28115018; PMCID: PMC5260012.
16. Aspinal F, Ledger J, Jasim S, et al. Implementation of the national Getting it Right First Time orthopaedic programme in England: a qualitative case study analysis BMJ Open 2023;13:e066303. doi: 10.1136/bmjopen-2022-066303
17. Getting It Right First Time. Effective Hip and Knee Replacement Pathway, 2020. Available from: https://gettingitrightfirsttime.co.uk/wp-content/uploads/2020/08/GIRFT-Hip-and-Knee-replacement-pathway-May-2020-003.pdf Accessed April 29, 2021.

For peer review only

For peer review only - http://bmjopen.bmj.com/site/about/guidelines.xhtml

18. Van Citters AD, Fahlman C, Goldmann DA, et al. Developing a pathway for high-value, patient-centered total joint arthroplasty. *Clin Orthop Relat Res.* 2014;472(5):1619-1635. doi:10.1007/s11999-013-3398-4
19. Marshall M, Gregory S, & Bullard E, Quality Patient Referrals. *Royal College of General Practitioners* 2018 Available from: https://www.rcgp.org.uk/getmedia/a83fc11f-dd08-4cdd-aefd-b647b03c5611/RCGP-referral-management-feb-2018.pdf
20. Goldsmith LJ, Suryaprakash N, Randall E et al. The importance of informational, clinical, and personal support in patient experience with total knee replacement: a qualitative investigation. *BMC Musculoskelet Disord.* 2017 Dec 24; 18 (1):127. doi: 10.1186/s12891-017-1474-8.
21. Conner-Spady BL, Marshall DA, Hawker GA, et al. You'll know when you're ready: a qualitative study exploring how patients decide when the time is right for joint replacement surgery. *BMC Health Serv Res*. 2014; 14:454. doi: 10.1186/1472-6963-14-454.
22. Sjöling M, Norbergh K-G, Malker H et al. What information do patients waiting for and undergoing arthroplastic surgery want? Their side of the story. *J Orthop Nurs*. 2006;10:5–14
23. Webster F, Perruccio AV, Jenkinson R et al. Where is the patient in models of patient-centred care: a grounded theory study of total joint replacement patients. *BMC Health Serv Res.* 2013 Dec 23;13:531. doi: 10.1186/1472-6963-13-531.
24. Carr T, Teucher UC, Casson AG. Time while waiting: patients' experiences of scheduled surgery. Qual Health Res. 2014 Dec;24(12):1673-85. doi: 10.1177/1049732314549022. Epub 2014 Sep 5.
25. Doyle C, Lennox L, Bell DA. Systematic review of evidence on the links between patient experience and clinical safety and effectiveness. *BMJ Open* 2013;3:e001570. doi: 10.1136/bmjopen-2012-001570
26. Bate P, Robert G. Experience-based design: from redesigning the system around the patient to co-designing services with the patient. *BMJ Quality & Safety* 2006;15:307-310.
27. Barratt H, Hutchings A, Pizzo E et al. Mixed methods evaluation of the Getting it Right First Time programme in elective orthopaedic surgery in England: an analysis from the National Joint Registry and Hospital Episode Statistics. *BMJ Open* 2022;0:e058316. doi:10.1136/bmjopen-2021-058316.
28. Kitzinger J. Qualitative research: Introducing focus groups. BMJ 1995;311:299
29. Focus Group: A Practical Guide for Applied Research. 5th Edition. Richard A. Krueger and Mary Anne Casey. 2015. Sage Publishing ISBN: 978-1-4833-6524-4.
30. Jeffery AE, Wylde V, Blom AW et al. 'It's there and I'm stuck with it': Patients' experiences of chronic pain following total knee replacement surgery. *Arthritis Care Res.* 2011;63:286–92
31. Ledger J, Mehta R, Jasim S et al. O12 Democratising the research process: reflections on the co-production of focus groups. *BMJ Open*, 2019, *9*(Suppl 1), A5–A5. https://doi.org/10.1136/bmjopen-2019-qhrn.12
32. NHS England. Transforming musculoskeletal and orthopaedic elective care services: rethinking referrals (NHSE), 2017. Available from: https://www.england.nhs.uk/wp-content/uploads/2017/11/msk-orthopaedic-elective-care-handbook-v2.pdf Accessed 1 May 2020
33. Brod M, Tesler LE, Christensen TL. Qualitative research and content validity: developing best practices based on science and experience. *Qual Life Res* 2009; 18: 1263–78.
34. Gale NK, Heath G, Cameron E et al. Using the framework method for the analysis of qualitative data in multi-disciplinary health research. *BMC Med Res Methodol* 2013, 13, 117. https://doi.org/10.1186/1471-2288-13-117

For peer review only - http://bmjopen.bmj.com/site/about/guidelines.xhtml

35. British Orthopaedic Association, 2020. NICE Trauma and Orthopaedic Guidelines. Available from: https://www.boa.ac.uk/standards-guidance/nice-trauma-and-orthopaedic-guidelines.html Accessed 15th March 2023
36. Mota REM, Tarricone R, Ciani, O et al. Determinants of demand for total hip and knee arthroplasty: a systematic literature review. BMC Health Serv Res,2012. 12, 225. https://doi.org/10.1186/1472-6963-12-225
37. Sandelowski M. Sample size in qualitative research. Research in Nursing & Health. 1995;18(2):179-183)
38. Punnoose A, Claydon-Mueller LS, Weiss O, Zhang J, Rushton A, Khanduja V. Prehabilitation for Patients Undergoing Orthopedic Surgery: A Systematic Review and Meta-analysis. JAMA Netw Open. 2023;6(4):e238050. doi:10.1001/jamanetworkopen.2023.8050
39. Grocott MP. Pathway redesign: putting patients ahead of professionals. Clin Med (Lond). 2019;19(6):468-472. doi:10.7861/clinmed.2019-0292
40. Carr A, Smith J A, Camaradou J et al. Growing backlog of planned surgery due to covid-19. BMJ 2021;372:n339. doi:10.1136/bmj.n339
41. McCall M, Peebles M, Sandiford NA: Cancellation of elective orthopaedic procedures is not a benign practice and is often preventable. N Z Med J 2021;134:70-78.
42. Caesar U, Karlsson L, Hamrin Senorski E, Karlsson J, Hansson-Olofsson E: Delayed and cancelled orthopaedic surgery; are there solutions to reduce the complex set of problems? A systematic literature review. Int J Clin Pract 2021;75:e14092.

For peer review only

Figure 1. Eligibility criteria

Figure 2. Patient pathway diagram

For peer review only

For peer review only - http://bmjopen.bmj.com/site/about/guidelines.xhtml

Figure 1: Eligibility criteria

All groups:

- Understand and speak English well enough to be able to give informed consent and participate in a group discussion
- Cognitively able to give informed consent and participate in a group discussion
- Not on a waiting list for total joint arthroplasty (TJA) (in case discussions affect their willingness to undertake TJA surgery)
- Aged over 18 years

Patient groups:

- Undergone elective TKA or THA between March 2016 and March 2018 (to minimise recall bias)
- Received **only** unplanned THA or TKA (because this is a different pathway)

Public group:

- Aged 60 years or older
- have **not** had TJA, but are willing to discuss NHS services to treat joint problems from a public perspective

For peer review only

The Patient Journey: Replacement Surgery

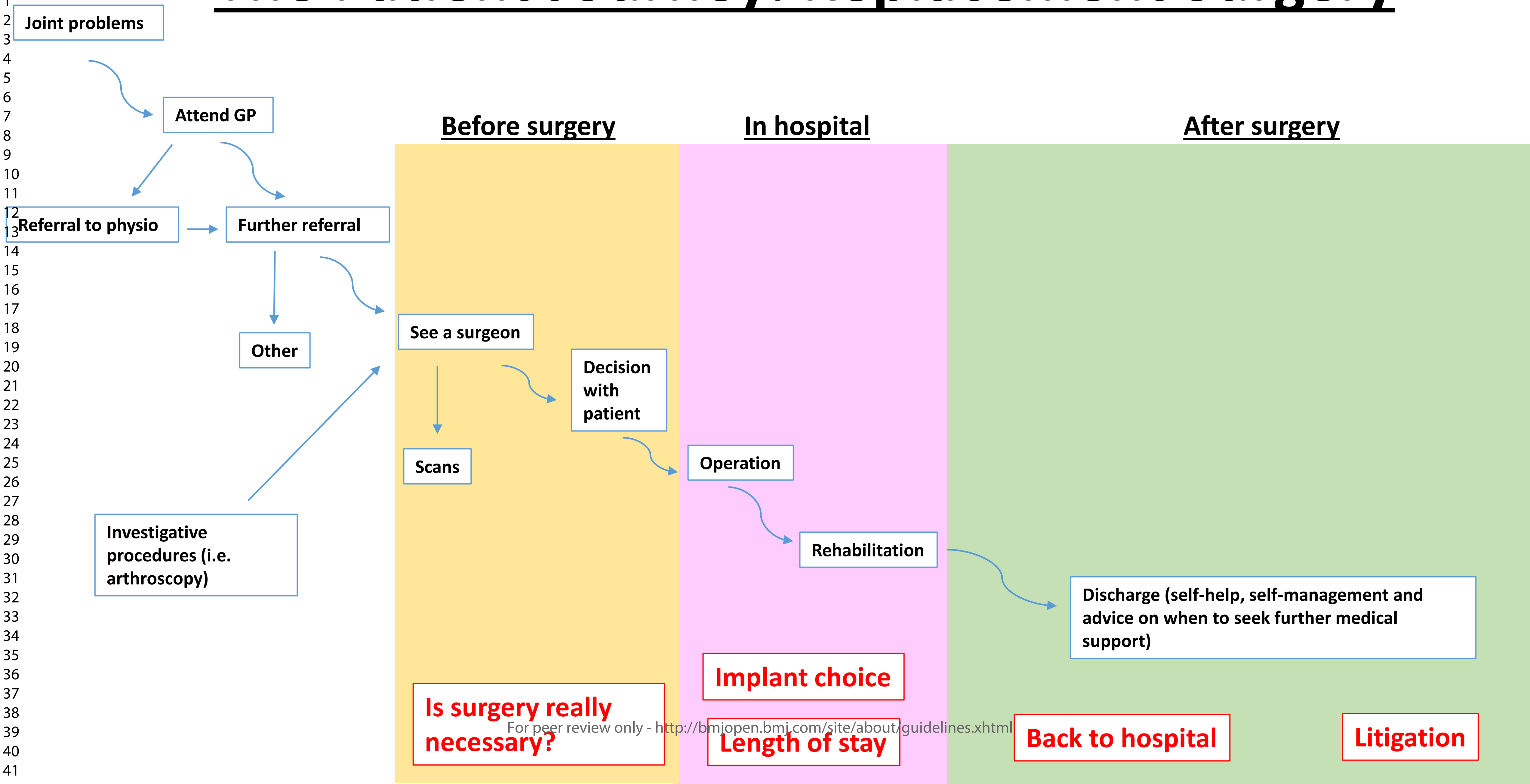

SUPPLEMENTAL MATERIAL

Online Supplementary File 1: SUMMARY TOPIC GUIDE 1 – GROUPS 1 & 2 (PATIENTS)

SECTION 1: EXPLORING PRACTICE: BEFORE, DURING AND AFTER SURGERY

Looking at this picture of the patient journey, we want to find out what was important to you at different stages:

1. **Before surgery** (orange box):

   At the point at which you were deciding whether to have a hip / knee replacement operation, what was important for you to know about?

2. **In hospital** (pink box):

   Based on your experience of having planned joint surgery in the NHS:

3. **After surgery** (green box):

   Once at home and following surgery, did you need to contact the NHS hospital where you were treated for any reason?

SECTION 2: EXPLORING GIRFT PROGRAMME PRIORITIES

4. In the presentation, we talked about how the GIRFT programme is trying to help hospitals across England provide similar 'results' for all patients who undergo planned orthopaedic surgery (e.g. to improve their life and reduce pain).

5. Are there any GIRFT programme priorities that were covered in the presentation today that we have not discussed and are also important to you?

6. Is there anything else important to you about joint surgery in the NHS, different from the GIRFT programme priorities and that we haven't talked about today?

SECTION 3: CLOSING COMMENTS

7. Finally, if you could give some feedback to the people leading this programme – what might you say?

For peer review only - http://bmjopen.bmj.com/site/about/guidelines.xhtml

Online Supplementary File 2: SUMMARY TOPIC GUIDE 2 – GROUP 3 (PUBLIC INDIVIDUALS)

SECTION 1: EXPLORING PRACTICE: BEFORE, DURING AND AFTER SURGERY

Looking at this picture of the patient journey, we want to find out what is important to you at different stages:

1. **Before surgery** (orange box):

   Scenario: Your GP has recommended that you see an orthopaedic surgeon, as you might need joint replacement surgery (e.g. a hip or knee replacement)

2. **After surgery** (green box):

   Scenario: Imagine you are at home after your hip/ knee replacement operation, and you become concerned about your recovery:

SECTION 2: EXPLORING GIRFT PROGRAMME PRIORITIES

3. In the presentation, we talked about how the GIRFT programme is trying to help hospitals across England provide similar 'results' for all patients who undergo planned orthopaedic surgery (e.g. to improve their life and reduce pain).

4. Are there any GIRFT programme priorities that were covered in the presentation today that we have not discussed and are also important to you?

5. Is there anything else important to you about joint surgery in the NHS, different from the GIRFT programme priorities and that we haven't talked about today?

SECTION 3: CLOSING COMMENTS

6. Finally, if you could give some feedback to the people leading this programme – what might you say?

For peer review only

Online Supplementary File 3: DETAILED STATEMENT FOR REFLEXIVITY

Reflexivity relates to acknowledgment of sensitivity to the ways in which the researchers and the research process may have shaped the data collected, including the role of prior assumptions and experience.[1]

Wider context of the research

Each focus group began with a short presentation explaining the context of the wider 'Getting It Right First Time' (GIRFT) [2] programme in elective orthopaedic surgery in England, and the programme priorities, to ensure all participants had a minimum level of knowledge. During analysis and interpretation, this pre-information was considered, and the possibility of how this knowledge might have shaped what was said.

Professional background of the research team

Members of the research team involved in fieldwork considered the ways in which their interactions with participants might be influenced by their own professional background, experiences, and prior assumptions. Co-facilitators (SJ and JL) were both academic research fellows from non-clinical backgrounds, the patient advisor (RM) who also co-facilitated focus groups had a non-clinical background but had previous experience of non-orthopaedic surgery. The observers (FA and HB) were both senior academic researchers who had clinical backgrounds. During our analysis and interpretation stages we considered whether knowing about our professional backgrounds could have impacted on participants' willingness to openly talk and share their experiences.

Potential for harm or distress

Members of the research team involved in fieldwork were sensitive to the possibility that focusing on the research topic (i.e. recalling previous surgical / in-hospital experiences) could potentially provoke anxiety in the focus group participants concerning the disclosure of adverse events. At the start of each focus group, co-facilitators (RM, SJ, JL) ensured that focus group participants could step out at any time or reveal or disclose as much of their previous experiences and perspectives as they felt comfortable with. At the end of each focus group, the research team stayed behind in the room to ensure that participants had the opportunity to seek support if they were feeling distressed by their participation. No focus group participants expressed such concerns or appeared to be distressed or uneasy.

Collaboration in knowledge production

Collaborative research is valued for its ability to bring together multiple researchers with distinctive and specialist perspectives to tackle large or complex research problems. There was a strong commitment within the research team from the outset to work collaboratively in the collection,

For peer review only - http://bmjopen.bmj.com/site/about/guidelines.xhtml

analysis, interpretation and reporting of the qualitative data, though individual involvement with the various stages of the research process varied as necessary. Team members closely involved in fieldwork (SJ, JL, RM, FA, HB) met frequently on separate occasions to discuss the progress of fieldwork and reflect on data collection. Meetings intensified during the initial stages of data collection and early analysis, when the topic guides and focus group materials were being produced and piloted, and themes and codes were beginning to be identified. At these crucial stages, input was sought from a senior member of the research team with extensive experience of qualitative research (NJF). In the final stages of analysis, input was sought from another senior member of the research team to sense check the coherence, truthfulness, and accuracy of the findings (RR). This resulted in an analytic strategy that was informed by insights from team members with a broad understanding of the research field and methodological issues, and those with field-based contextual and experiential understanding.

For peer review only

REFERENCE LIST

1. Jootun D, McGhee G, Marland G R. Reflexivity: promoting rigour in qualitative research. *Nurs stand.* 2009;23(23): 42-47.
2. Briggs T. Getting it Right First Time: improving the quality of orthopaedic care within the National Health Service in England, 2012. London, United Kingdom: British Orthopaedic Association.

For peer review only

For peer review only - http://bmjopen.bmj.com/site/about/guidelines.xhtml

Standards for Reporting Qualitative Research (SRQR)*

http://www.equator-network.org/reporting-guidelines/srqr/

Title and abstract

	Page/line no(s).
**Title** - Concise description of the nature and topic of the study Identifying the study as qualitative or indicating the approach (e.g., ethnography, grounded theory) or data collection methods (e.g., interview, focus group) is recommended	1
**Abstract** - Summary of key elements of the study using the abstract format of the intended publication; typically includes background, purpose, methods, results, and conclusions	2

Introduction

**Problem formulation** - Description and significance of the problem/phenomenon studied; review of relevant theory and empirical work; problem statement	4
**Purpose or research question** - Purpose of the study and specific objectives or questions	4

Methods

**Qualitative approach and research paradigm** - Qualitative approach (e.g., ethnography, grounded theory, case study, phenomenology, narrative research) and guiding theory if appropriate; identifying the research paradigm (e.g., postpositivist, constructivist/ interpretivist) is also recommended; rationale**	5
**Researcher characteristics and reflexivity** - Researchers' characteristics that may influence the research, including personal attributes, qualifications/experience, relationship with participants, assumptions, and/or presuppositions; potential or actual interaction between researchers' characteristics and the research questions, approach, methods, results, and/or transferability	6 & Supplement
**Context** - Setting/site and salient contextual factors; rationale**	6
**Sampling strategy** - How and why research participants, documents, or events were selected; criteria for deciding when no further sampling was necessary (e.g., sampling saturation); rationale**	5
**Ethical issues pertaining to human subjects** - Documentation of approval by an appropriate ethics review board and participant consent, or explanation for lack thereof; other confidentiality and data security issues	6
**Data collection methods** - Types of data collected; details of data collection procedures including (as appropriate) start and stop dates of data collection and analysis, iterative process, triangulation of sources/methods, and modification of procedures in response to evolving study findings; rationale**	6

For peer review only

For peer review only - http://bmjopen.bmj.com/site/about/guidelines.xhtml

**Data collection instruments and technologies** - Description of instruments (e.g., interview guides, questionnaires) and devices (e.g., audio recorders) used for data collection; if/how the instrument(s) changed over the course of the study	6
**Units of study** - Number and relevant characteristics of participants, documents, or events included in the study; level of participation (could be reported in results)	5
**Data processing** - Methods for processing data prior to and during analysis, including transcription, data entry, data management and security, verification of data integrity, data coding, and anonymization/de-identification of excerpts	6
**Data analysis** - Process by which inferences, themes, etc., were identified and developed, including the researchers involved in data analysis; usually references a specific paradigm or approach; rationale**	6
**Techniques to enhance trustworthiness** - Techniques to enhance trustworthiness and credibility of data analysis (e.g., member checking, audit trail, triangulation); rationale**	6

Results/findings

**Synthesis and interpretation** - Main findings (e.g., interpretations, inferences, and themes); might include development of a theory or model, or integration with prior research or theory	7-12
**Links to empirical data** - Evidence (e.g., quotes, field notes, text excerpts, photographs) to substantiate analytic findings	7-12

Discussion

**Integration with prior work, implications, transferability, and contribution(s) to the field** - Short summary of main findings; explanation of how findings and conclusions connect to, support, elaborate on, or challenge conclusions of earlier scholarship; discussion of scope of application/generalizability; identification of unique contribution(s) to scholarship in a discipline or field	13
**Limitations** - Trustworthiness and limitations of findings	3 & 13

Other

**Conflicts of interest** - Potential sources of influence or perceived influence on study conduct and conclusions; how these were managed	14
**Funding** - Sources of funding and other support; role of funders in data collection, interpretation, and reporting	14

*The authors created the SRQR by searching the literature to identify guidelines, reporting standards, and critical appraisal criteria for qualitative research; reviewing the reference lists of retrieved sources; and contacting experts to gain feedback. The SRQR aims to improve the transparency of all aspects of qualitative research by providing clear standards for reporting qualitative research.

For peer review only

**The rationale should briefly discuss the justification for choosing that theory, approach, method, or technique rather than other options available, the assumptions and limitations implicit in those choices, and how those choices influence study conclusions and transferability. As appropriate, the rationale for several items might be discussed together.

Reference:

O'Brien BC, Harris IB, Beckman TJ, Reed DA, Cook DA. **Standards for reporting qualitative research: a synthesis of recommendations.** *Academic Medicine*, Vol. 89, No. 9 / Sept 2014 DOI: 10.1097/ACM.0000000000000388

For peer review only
